# Etiology matters – Genomic DNA Methylation Patterns in Three Rat Models of Acquired Epilepsy

**DOI:** 10.1038/srep25668

**Published:** 2016-05-09

**Authors:** Konrad J. Dębski, Asla Pitkanen, Noora Puhakka, Anna M. Bot, Ishant Khurana, KN Harikrishnan, Mark Ziemann, Antony Kaspi, Assam El-Osta, Katarzyna Lukasiuk, Katja Kobow

**Affiliations:** 1 Laboratory of Epileptogenesis, Department of Molecular and Cellular Neurobiology, Nencki Institute of Experimental Biology, Polish Academy of Sciences, Warsaw, Poland; 2 Epilepsy Research Laboratory, A. I. Virtanen Institute for Molecular Sciences, University of Eastern Finland, PO Box 1627, FIN-70211 Kuopio, Finland; 3 Epigenetics in Human Health and Disease, Baker IDI Heart and Diabetes Institute, The Alfred Medical Research and Education Precinct, Melbourne, VIC, Australia; 4 Department of Neuropathology, University Hospital Erlangen, Schwabachanlage 6, 91054 Erlangen, Germany

## Abstract

This study tested the hypothesis that acquired epileptogenesis is accompanied by DNA methylation changes independent of etiology. We investigated DNA methylation and gene expression in the hippocampal CA3/dentate gyrus fields at 3 months following epileptogenic injury in three experimental models of epilepsy: focal amygdala stimulation, systemic pilocarpine injection, or lateral fluid-percussion induced traumatic brain injury (TBI) in rats. In the models studies, DNA methylation and gene expression profiles distinguished controls from injured animals. We observed consistent increased methylation in gene bodies and hypomethylation at non-genic regions. We did not find a common methylation signature in all three different models and few regions common to any two models. Our data provide evidence that genome-wide alteration of DNA methylation signatures is a general pathomechanism associated with epileptogenesis and epilepsy in experimental animal models, but the broad pathophysiological differences between models (i.e. pilocarpine, amygdala stimulation, and post-TBI) are reflected in distinct etiology-dependent DNA methylation patterns.

According to the WHO, 60 million individuals have epilepsy worldwide, and in 30% of patients, available treatments are ineffective or cause life-compromising adverse events^1^,^2^. Identifying novel mechanisms that lead to the development of epilepsy (epileptogenesis) and its progression culminating in drug-refractory epilepsy is a major unmet medical need^3^,^4^,^5^. Recent evidence indicates a spectrum of dysfunctional epigenetic mechanisms that occur during epileptogenesis, including acute changes in DNA methylation, histone modifications, and microRNA expression^6^. Kobow *et al*. demonstrated epilepsy associated changes in methylation of the reelin promoter is associated with granule cell dispersion in epileptic subjects^7^. Epigenetic changes have since been reported by several laboratories in different epilepsy models (for review, see^6^,^8^,^9^). These studies have suggested that in epilepsies triggered by traumatic brain injury (TBI) or status epilepticus (SE), epigenetic changes are associated with inherited and acquired etiological factors and complex reorganization of neuronal and glial networks during epileptogenesis. Because the etiologies of epilepsy vary greatly, a critical question is whether there are general mechanisms that could be targeted to design treatments for epileptogenesis after different types of brain insults.

We have hypothesized that epileptogenesis is accompanied by genomic methylation changes. To assess whether hippocampal DNA methylation patterns are common between different epilepsy models, we investigated three animal models of acquired epilepsy: focal SE (electrical stimulation of the amygdala), systemic SE (pilocarpine), and TBI in rats. The models share some qualitative similarities in hippocampal circuitry reorganization believed to lead to the development of epilepsy such as synaptic, axonal and dendritic plasticity, suggesting common molecular mechanisms of epileptogenesis.

In the present study, genomic DNA methylation patterns distinguished controls from injured animals pointing at a general pathomechanism associated with epileptogenesis. Nevertheless, the broad pathophysiological differences between models (i.e. pilocarpine, amygdala stimulation, and post-TBI) were reflected in distinct etiology-dependent epigenetic and gene expression signatures.

## Results

**Figure 1:**
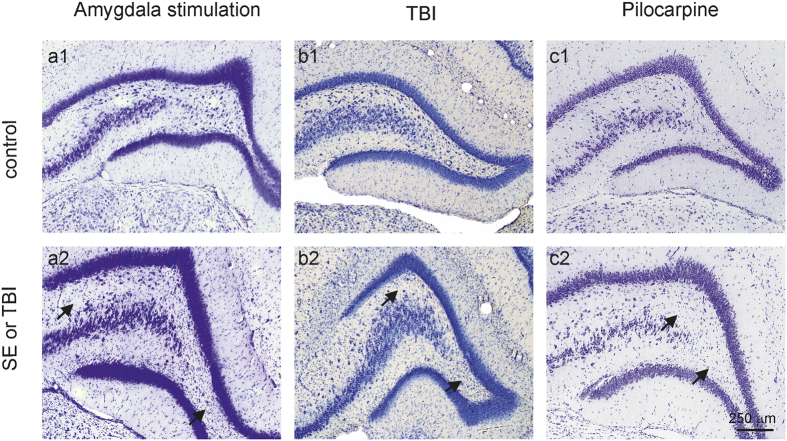
Pattern of neurodegeneration in three models of acquired epilepsy. Nissl-stained sections presenting dentate gyrus of animals from amygdala stimulation **(a2)**, pilocarpine **(b2)** and TBI **(c2)** model and respective controls **(a1,b1,c1)**. Arrows indicate neuronal loss.

We hypothesized that epileptogenesis is accompanied by specific gene expression changes mediated by DNA methylation. To investigate this we compared DNA methylation in three experimental models of epilepsy. We used methyl-capture and massive parallel sequencing (Methyl-Seq) of hippocampal tissue obtained from rats at 3 months following focal amygdala stimulation, systemic pilocarpine injection, or lateral fluid-percussion-induced TBI with corresponding sham-treated controls. Genome-wide methylation patterns in the pilocarpine model have been previously described in detail^10^. Here we reanalyzed the publicly available data (NCBI Gene Expression Omnibus accession number GSE50080) and integrated these with our results. To analyze the biological relevance of methylation changes, we examined changes in gene expression using mRNA sequencing in the same samples (mRNA-Seq). We focused on integrative analysis of datasets obtained from the three models, which had distinct pathology in the hippocampus (Fig. 1).

### Genomic DNA methylation signatures distinguish models of acquired epilepsy from controls

**Figure 2:**
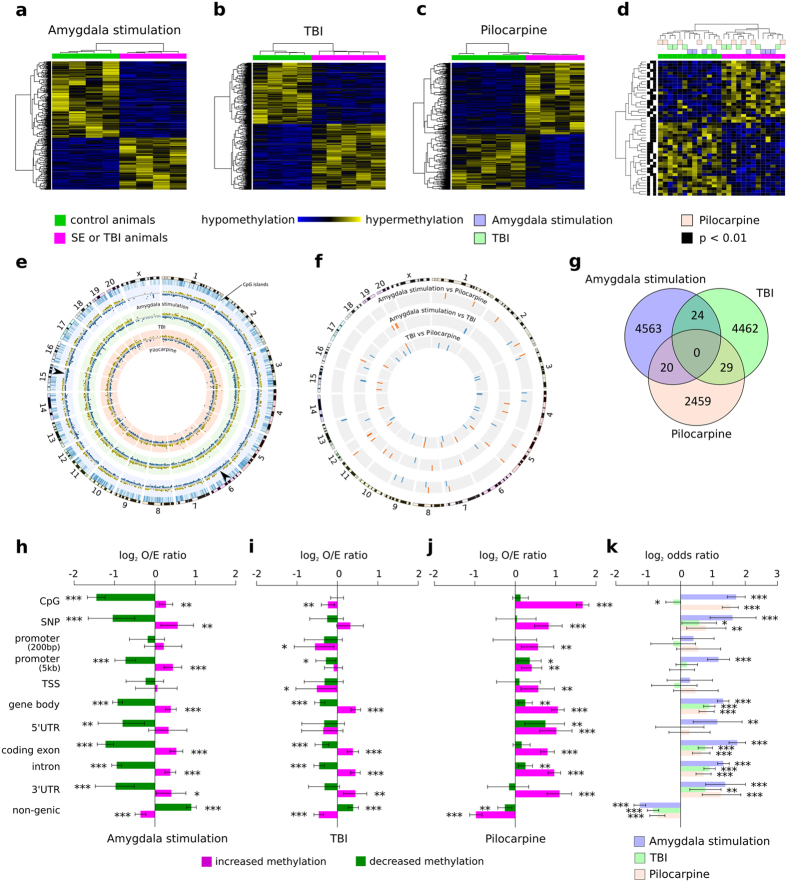
Differentially methylated regions and their distribution in three models of epilepsy. (**a–c**) - Unsupervised hierarchical cluster analysis of differential methylation in **(a)** amygdala stimulation, **(b)** TBI, and **(c)** pilocarpine models. Hypermethylation – yellow, hypomethylation – blue, green – control, magenta - SE/TBI. (**d**) - Heatmap summarizing overlapping regions with consistent change in DNA methylation in at least 2 of 3 models of epilepsy (p < 0.01). Hypermethylation – yellow, hypomethylation – blue, green – control, magenta - SE/TBI. (**e**) - Rat genome ideogram summarizing the probability of increased (yellow) or decreased (blue) DNA methylation in injured *vs.* control animals in three animal epilepsy models. Outer ring represents chromosomes. Inner rings indicate location of methylation events in each model (amygdala stimulation - light blue, TBI - light green, pilocarpine - light red, hypermethylation – yellow, hypomethylation – blue). Distance from black line in the middle of each ring represents increasing probability of methylation change (presented as -log10 of p-value). Arrowheads indicate areas in chromosomes 6 and 15 that lacked increased methylation in the amygdala stimulation model. (**f**) - Rat genome ideogram summarizing DNA methylation changes common for pairs of models (inner gray rings). Outer ring represents chromosomes. Red bars - common increased methylation events, blue bars - common decreased methylation events. (**g**) - Venn diagram presenting differentially methylated DNA regions detected in each model of epilepsy and regions overlapping between models with change in methylation to the same direction. (**h–j**) - Genomic distribution of DNA methylation changes in **(h)** amygdala stimulation, **(i)** TBI, and **(j)** pilocarpine models. Frequency of observed methylation changes compared with non-differentially methylated regions (p < 0.01), with upper and lower 95% confidence intervals for different genomic features. Magenta - increased methylation, green - decreased methylation, O/E - observed/expected ratio, CpG - CpG islands, SNP - single nucleotide polymorphism, TSS - transcriptional start site, UTR - untranslated region. (**k**) - Ratio of increased to decreased methylation events across genomic features for each epilepsy model with upper and lower 95% confidence intervals. Blue, green and light red bars represent the focal amygdala stimulation, TBI, and systemic pilocarpine models, respectively. ^*^p < 0.05, **p < 0.01, ***p < 0.001 (two-sided Fisher’s exact test).

Pairwise comparisons of global DNA methylation was performed for models of epilepsy (sham versus injury) separately to allow to concentrate on injury/epilepsy related alterations and omit those related to e.g. strain, living conditions or handling procedures in different labs. We observed strong differences in methylation patterns between control and injured animals in all three epilepsy models (Fig. 2a–c). Unsupervised clustering of differentially methylated regions (p < 0.01 between control and epileptic animals) in the amygdala stimulation (Fig. 2a), TBI (Fig. 2b), and pilocarpine (Fig. 2c) model suggests DNA methylation distinguishes injured epileptogenic hippocampal tissue in each model. We identified 2741 hypomethylated and 1866 hypermethylated regions in epileptic tissue from the amygdala stimulation model compared to controls; 1121 hypomethylated and 1452 hypermethylated regions in pilocarpine treated epileptic animals over healthy controls; and 2171 hypomethylated and 2344 hypermethylated regions in the TBI model as compared to controls.

We conclude alterations to genomic DNA methylation provide a general pathomechanism associated with epileptogenesis.

### DNA methylation events commonly shared between epilepsy models

To investigate commonalities between animal models, we intersected differentially methylated regions from all three datasets. Regions overlapping by at least one base pair and with methylation changes occurring in the same direction were defined as common. The heatmap presented in Fig. 2d shows results from unsupervised clustering (using z-scores calculated separately for each model, p < 0.01) of those regions with changes in methylation common to two models. Clusters distinguish all control animals from injuries, regardless of their genetic background and etiology. However, it has to be noted that the majority of observed alterations in genomic DNA methylation in each model were related to the initial precipitating injury and subsequent epileptogenic process.

We further mapped differentially methylated regions to the rat genome (RN5) using Circos, as shown in Fig. 2e. Increased (yellow) and decreased (blue) probability of methylation change was assigned to each chromosome (as -log10 of p-value). In all models methylation events were distributed throughout the genome, excluding chromosome X. Some differences between models could be observed. In the focal amygdala stimulation model, some areas did not exhibit increased methylation, *e.g.* chromosome 6 and 15, in contrast to the systemic pilocarpine and TBI models (black arrowheads in Fig. 2e). More focused analysis identified only twenty-four differentially methylated regions to be common to focal amygdala stimulation and TBI models, 20 were common to focal amygdala stimulation and systemic pilocarpine models, and 29 were common to TBI and systemic pilocarpine models (Fig. 2d,f,g). Two regions in promoters, 15 in gene bodies, and 10 in non-genic areas were common to the focal amygdala stimulation and TBI models. Four regions in promoters, 9 in gene bodies, and 11 non-genic areas were common to the focal amygdala stimulation and systemic pilocarpine models. Two regions in promoters, 16 in gene bodies, and 16 in non-genic areas were common to the TBI and systemic pilocarpine models. No DNA methylation events were common to all three models. Few regions displayed methylation changes in the opposite direction: 9 between the amygdala stimulation and TBI; 8 between amygdala stimulation and systemic pilocarpine; and 15 between systemic pilocarpine and TBI.

We conclude that the localization of hypo- and hypermethylation is primarily model-specific.

### Genomic distribution of DNA methylation in the three epilepsy models

To determine whether alterations in methylation status occur preferentially in specific genomic features, we compared the distribution of hypo- and hypermethylation events at CpG islands, single nucleotide polymorphisms (SNP), promoters (200 bp and 5 kb upstream of transcription start), transcription start sites (TSS), gene body (including 5′ and 3′ untranslated regions), 5′ untranslated regions (5′UTR), coding exons (protein coding part of exon), introns, 3′UTR, and non-genic regions. Figure 2h–j show the mean frequency of methylation changes with upper and lower 95% confidence intervals for the described genomic features in all three models of acquired epilepsy. A preliminary analysis of the distribution of methylation changes in the pilocarpine-induced model of epilepsy was performed by Kobow *et al*.^10^. Here, we extended the list of evaluated genomic features.

We observe methylation changes at genomic features were predominantly model-specific. For example, in the focal amygdala stimulation model, hypermethylation events were frequently observed in CpG islands, SNPs, promoters (5 kb), gene bodies, coding exons, introns, and 3′UTRs. In contrast, hypomethylation events occurred less frequently than expected in most genomic features, as shown by the negative odds ratios. Infact, non-genic regions exhibited a different profile in this model, with an increased probability of hypomethylation and decreased probability of hypermethylation (Fig. 2h).

In the TBI model, hypermethylation was primarily observed in gene bodies, coding exons, introns, and 3′UTRs and rarely affected CpG islands, promoters (200 bp), TSS, and non-genic regions. Hypomethylation occurred more frequently in non-genic regions and less frequently in promoters (5 kb), gene bodies, coding exons, and introns (Fig. 2i).

Re-analysis of the pilocarpine epilepsy model^10^ verified increased DNA methylation in most genomic features including 3′ and 5′UTRs, promoter sequences (200bp and 5 kb), as well as SNPs (Fig. 2j). Next, we compared altered methylation events at specific genomic features between models (Fig. 2k). We observed decreased hypermethylation in non-genic regions in the three models, suggesting an overlap. Hypermethylation was more frequent in SNPs, gene bodies, coding exons, introns, and 3′UTRs in the three models. Interestingly, an increase in methylation frequency in CpG islands was found in the focal amygdala stimulation and systemic pilocarpine models only. In the focal amygdala stimulation model, increased methylation frequency was detected in 5′UTRs and promoters (5 kb).

### Gene expression patterns differentiate control from injury-induced epilepsy models

**Figure 3:**
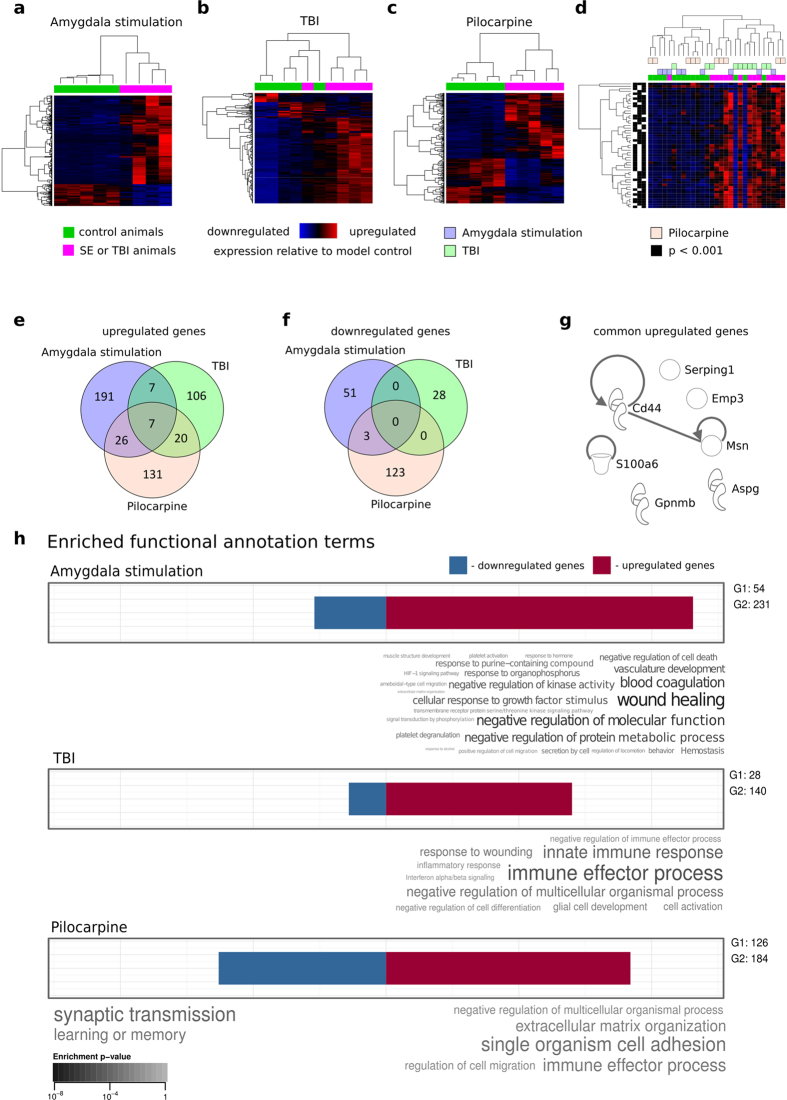
Differentially expressed genes over three models of TLE. (**a–c**) - Unsupervised hierarchical clustering of samples and transcripts according to differential expression profiles (upregulation - red, downregulation - blue, cut-off p < 0.001) for the amygdala stimulation (**a**), TBI (**b**) and pilocarpine model (**c**). Green bars on the top of the heatmaps refer to control and magenta bars to SE/TBI animals. Data from pilocarpine model were previously published by Kobow *et al*.^10^ and reanalyzed here as described in Methods section. (**d**) - Heatmap presenting genes with altered expression levels in at least two animal models (cut-off p < 0.001). Blue color indicates downregulation and red upregulation of gene expression in a given model of epilepsy. The blue bar on the top of the heatmap refers to control and the magenta bar to SE/TBI animals. Black shadings on the left side of the heatmap represent the significant differences. (**e–f**) - Venn diagrams presenting upregulated (**e**) or downregulated (**f**) genes common between models. (**g**) - Direct and indirect experimentally observed interaction between genes or gene products according to IPA. Note that interactions were found between Msn (moesin) and Cd44 (Cd44 antigen precursor), and that feedback interaction was observed for S100a6, Msn and Cd44. (**h**) - Word clouds presenting enrichment analysis of terms from the GO database from the Biological Process branch and terms from biological pathways databases (KEGG and Reactome) with gProfiler for each model. The length of blue and red bars over each word cloud are proportional to the number of down- and upregulated genes, respectively. The numbers on the right side of the bars (G1 and G2) represent the number of down- and upregulated genes, respectively. Word clouds under the blue bars describe enriched terms for downregulated genes whereas words under the red bars represent upregulated genes. Only significantly enriched terms (enrichment p < 0.01) are presented. The color of the font indicates the enrichment p-value. Darker colors indicate more significant enrichment. The font size is related to the significance of enrichment of a given term but only within a given cloud.

Pairwise comparisons of RNA-sequencing were performed for the amygdala stimulation model (Fig. 3a) which was similar to the pilocarpine model previously published by Kobow *et al*. (see Fig. 3 in Kobow *et al*.) and reanalyzed in this paper (Fig. 3c)^10^. In the TBI model samples were partially mixed. One out of five samples from injured animals was more similar in the expression pattern to control animals (Fig. 3b). Altogether we found 54 upregulated and 231 downregulated genes in the amygdala stimulation model, 140 upregulated and 28 downregulated genes following TBI, and 184 upregulated and 126 downregulated genes in the pilocarpine model. Lists of genes with altered expression are presented in the Supplementary Tables 1 and 2. In summary, RNA expression patterns can distinguish injured and control animals in all three models.

### Gene expression changes common to the three epilepsy models

When samples from all three experimental animal models were combined (z-score calculated separately for each model; genes with expression changes in the same direction for at least two models), unsupervised clustering of gene expression patterns separated most control from epileptic animals, although the separation was not complete (Fig. 3d). Some control samples (3 out of 15) clustered with injured animals, and some of injured animals (2 out of 14) clustered with control animals.

To determine which expression changes were common to more than one experimental model, we compared lists of differentially expressed genes (cut-off p <  0.001) from all three datasets. Fourteen upregulated and no downregulated genes were common to the amygdala stimulation and TBI models, 33 upregulated and 3 downregulated genes were common to the amygdala stimulation and pilocarpine models, and 27 upregulated and no downregulated genes were common to the TBI and pilocarpine models (Fig. 3e,f). Additionally, 7 upregulated genes were common to all three epilepsy models: Serping 1 [serpin peptidase inhibitor, clade G (C1 inhibitor), member 1], Emp3 (epithelial membrane protein 3), S100a6 (S100 calcium binding protein A6), Msn (moesin), Cd44 (CD44 molecule), Aspg (asparaginase), and Gpnmb [glycoprotein (transmembrane) nmb] (Fig. 3e). We conclude that the gene expression pattern is primarily model-specific, although some genes behave similarly in all three epilepsy models.

Next, we searched for any direct and indirect interactions between the 7 genes upregulated in all three epilepsy models or their protein product. Interactions were only observed between Msn and Cd44, whereas other genes are not functionally connected (Fig. 3g) suggesting that the genes upregulated are unlikely to underlie one metabolic process.

We also addressed the question if genes involved in epigenetic regulation of transcription are altered in any of studied models. We selected enzymes involved in DNA methylation (DNMTs, TETs), methyl-CpG binding domain proteins, histone methyltransferases, demethylases, acetyltransferases, deacetylases, and transcription factors having histone modifying properties. At a relaxed cut-off p < 0.01, changes in expression level were found for DOT1L (fold change 2.24; p < 0.001), Lysine (K)-Specific Demethylase 6B (KDM6B, fold change 3.302, p < 0.01), and PR Domain Containing 2, With ZNF Domain (PRDM2; fold change −1.26; p < 0.01) in the amygdala stimulation model, and for Enhancer Of Zeste 1 Polycomb Repressive Complex 2 Subunit (EZH1) in the pilocarpine model (fold change 1.69, p < 0.01). No changes were observed in genes involved in DNA methylation regulation. The increase in the expression of DNMT3A in pilocarpine model (1.64 fold; p = 0.040) did not reach our significance criteria.

### Functional annotations of genes differentially expressed in each epilepsy model

To determine whether genes differentially expressed in each model of epilepsy are a part of a common biological process or pathway, we performed enrichment analysis based on annotation terms from GO, KEGG and Reactome (Fig. 3h).

No significantly enriched terms were observed for downregulated genes in the amygdala stimulation and TBI models. There were two enriched terms for genes downregulated in the systemic pilocarpine model: synaptic transmission and learning or memory (Fig. 3h).

There were 25 enriched terms for upregulated genes in the amygdala stimulation model. The ten most enriched terms in order of significance, were as follows: wound healing, negative regulation of molecular function, blood coagulation, negative regulation of protein metabolic process, vasculature development, cellular response to growth factor stimulus, negative regulation of kinase activity, negative regulation of cell death, response to organophosphorus and hemostasis. The enriched terms for upregulated genes in the TBI model were as follows: immune effector process, innate immune response, negative regulation of multicellular organismal process, response to wounding, cell activation, glial cell development, negative regulation of cell differentiation, negative regulation of immune effector process, inflammatory response, and interferon alpha/beta signaling. In the systemic pilocarpine model, five enrichment terms characterizing upregulated genes were found: single organism cell adhesion, immune effector process, extracellular matrix organization, negative regulation of multicellular organismal process, and regulation of cell migration (Fig. 3h). Interestingly, most terms were model-specific and only a few occurred in more than one model. Immune effector process was detected in the TBI and systemic pilocarpine models, and negative regulation of molecular function was detected in the amygdala stimulation and systemic pilocarpine models. Additionally, extracellular organization process, which was the second most significantly enriched in the systemic pilocarpine model, was the least significant of all 25 enriched terms in the amygdala stimulation model.

These data imply that different biological processes predominate in each chronic epilepsy model, although some functional similarities can be observed.

### Correlation of aberrant DNA methylation and gene expression in the three epilepsy models

To understand the relationships between genomic methylation and gene expression in different models of chronic epilepsy in rats, we integrated DNA methylation with gene expression data derived from the same hippocampal specimens. Preliminary analyses of the relationships between methylation changes and mRNA expression for systemic pilocarpine model have been previously published^10^. Here, we extend these results by considering methylation events in 3′ UTRs, 5′ UTRs, 5 kb promoter and CGI in the 5 kb promoter. Gene expression profiling identified 404 transcripts that were differentially expressed in focal amygdala stimulation, 229 transcripts in TBI, and 313 transcripts in pilocarpine models (cut-off p < 0.001).

**Figure 4:**
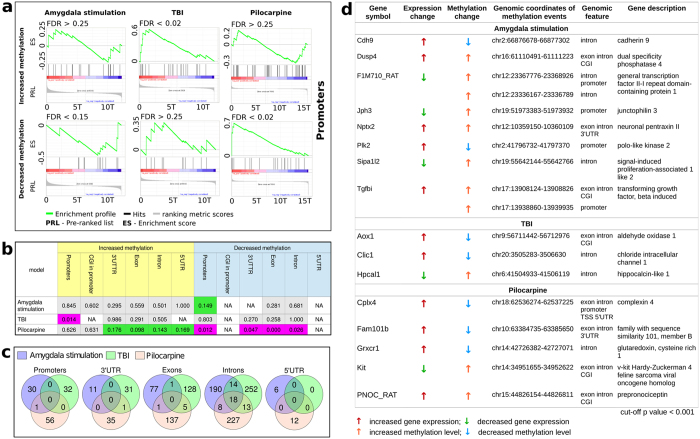
Correlation between methylation and gene expression. (**a**) - Examples of GSEA enrichment profiles of association between gene expression and alterations in promoter methylation, A strong association (FDR < 0.25 was considered significant) was observed between increased promoter methylation and gene activation in the TBI model, between decreased promoter methylation and gene repression in the focal amygdala stimulation model, and between decreased promoter methylation and gene activation in the systemic pilocarpine model. ES - enrichment score, PRL - pre-ranked list, FDR - false discovery rate. (**b**) - Gene set enrichment analysis (GSEA) presenting associations between gene expression and methylation changes in different genomic features in three epilepsy models. The results are presented as false discovery rates of nominal p-values of gene sets of increased or decreased methylation events at promotors (5 kb upstream of transcription start), CpG islands in promoters (CGI in promoter), TSSs, 3′UTRs, exons, introns and 5′UTRs against the rank of mRNA-Seq data from the same samples. Cell background color representations are as follows: gray – non-significant association, green - significant association with decreased gene expression, magenta - significant association with increased gene expression (FDR < 0.25 was considered as significant). NA - analysis not available due to missing differentially methylated regions in gene sets. (**c**) - Venn diagrams showing overlaps between genes and differentially methylated DNA regions used in GSEA. (**d**) - List of differentially expressed genes (cut-off p < 0.001), in which methylation changes occurred in gene body or promoter regions in each model. Magenta and green arrows indicate increased and decreased levels of mRNA, respectively. Orange and blue arrows indicate increased and decreased methylation events, respectively, which occurred in promoter regions, TSSs, exons, introns, 3′UTRs, 5′UTRs and CGIs (CpG islands).

As shown in Fig. 4a,b in the focal amygdala stimulation model decreased methylation in promoters was linked to decreased gene expression. In contrast, the systemic pilocarpine model showed an inverse correlation linking decreased promoter methylation with increased gene expression, whereas no association between decreased promoter methylation and gene expression was observed in the TBI model. However, increased methylation of promoters in the TBI model was linked to increased gene expression, whereas no such association was detected in the other two models. No associations between gene expression and methylation in genomic features, other than promoters were detected in the focal amygdala stimulation and TBI models (Fig. 4b). Only in the systemic pilocarpine model an association between increased methylation in 3′UTRs, exons, introns, and 5′UTRs and decreased gene expression was observed as well as between decreased methylation in 3′UTRs, exons, and introns and the increased gene expression, thereby extending previous reports on this data set^10^.

To further investigate the influence of methylation on gene expression, we concentrated specifically on genes that exhibited altered methylation status in at least two models (Fig. 4c). In particular, we investigated promoters differentially methylated in all three models. Interestingly, 18 genes had introns that were differentially methylated in all three epilepsy models. Some common methylation events were observed when comparing epilepsy models in pairs, specifically 15 out of 32 genes with altered methylation in introns common to the focal amygdala stimulation model and TBI could be identified. Eleven exhibited increased methylation and four decreased methylation events (Supplementary Table 3). When comparing focal amygdala stimulation and systemic pilocarpine models, 9 methylation events occurred in the same regions in introns. Six exhibited increased methylation and three decreased methylation (Supplementary Table 3). Comparisons between TBI and systemic pilocarpine models revealed 16 common methylation events occurring in the same region in introns. Seven exhibited increased methylation and nine decreased methylation (Supplementary Table 3).

To identify individual relationships between DNA methylation changes and gene expression levels in each model of epilepsy, we generated a list of differentially expressed genes (cut-off p < 0.001) for which differentially methylated regions of DNA (cut-off p < 0.001) were identified in promoters (defined as 5 kb upstream of TSS) or gene bodies. As shown in Fig. 4d, in the focal amygdala stimulation model 8 genes, in which promoters or gene bodies were differentially methylated, were differentially expressed. Changes in promoter methylation were accompanied by changes in mRNA levels for F1M710_RAT (general transcription factor II-I repeat domain-containing protein 1), Jph3 (junctophilin 3), Plk2 (polo-like kinase 2), and Tgfbi (transforming growth factor, beta induced). Changes in mRNA expression were accompanied by changes in methylation status in introns for Cdh9 (cadherin 9), Dusp4 (dual specificity phosphatase), F1M710_RAT, Nptx2 (neuronal pentraxin II), Sipa1|2 (signal-induced proliferation-associated 1 like 2), and Tgfbi. In the TBI model, we identified 3 genes in which changes in mRNA expression were accompanied by changes in methylation in introns Aox1 (aldehyde oxidase 1); Clic 1 (chloride intracellular channel 1); Hpcal1 (hippocalcin-like 1)]. In the systemic pilocarpine model, changes in the methylation status of introns in Cplx4 (complexin 4), Fam101b (family with sequence similarity 101, member B), Grxcr1 (glutaredoxin, cysteine rich 1), Kit (v-kit Hardy-Zukerman 4 feline sarcoma viral oncogene homolog), and PNOC_RAT (prepronociceptin) were accompanied by changes of mRNA levels. Moreover, changes in the methylation status of Cplx4 were also present in promoter and TSS. No genes with altered expression levels and methylation status were common to at least 2 models, indicating model specificity in this phenomenon.

## Discussion

This study investigated the generality of DNA methylation alterations in three experimental models of chronic epilepsy, representing a general pathomechanism regardless of etiology. In all injury models studied, DNA methylation status clearly distinguished controls from focal amygdala stimulation, systemic pilocarpine and TBI animals. The models were characterized by markedly increased methylation in gene bodies and hypomethylation in non-genic areas. However, analysis of the precise locations of methylation events in the genome did not identify regions with altered methylation common to all three models, and only a few regions were common to any two models. Modest associations between methylation in different genomic areas and alterations in gene expression were observed and appeared to be strongest in the systemic pilocarpine model.

Gene-specific as well as genome-wide alterations in DNA methylation have been previously indicated in epilepsy^7^,^10^,^11^,^12^,^13^,^14^,^15^. TLE patients with hippocampal sclerosis and animals with experimental epilepsy have been reported with increased DNA methyltransferase (DNMT) levels in the hippocampus^12^,^13^,^16^ and increased global DNA methylation^12^,^13^. In hippocampus sampled from rat systemic kainate model or patients with drug-refractory TLE, there is a clear predominance of increased methylation in gene promoters^13^,^15^. Finally, whole-genome next-generation sequencing of hippocampal tissue from chronically epileptic animals in the systemic pilocarpine model has revealed widespread alterations in methylation status throughout the genome with prevailing hypermethylation events in the gene body . Our findings extend these observation by showing that hypermethylation of gene bodies is a common feature in experimental models of epilepsy and possibly related to brain hyperexcitability. This hypothesis is supported by previous studies demonstrating that therapeutic adenosine augmentation or ketogenic diet decrease DNA methylation levels while attenuating epileptogenesis^10^,^13^. On the other hand, it has been shown that inhibition of DNMT at the time of initial insult in the rat kainic acid model increased excitatory postsynaptic potentials in hippocampal slices isolated at 14 d later, and caused a decrease in the latency to the first spontaneous seizure^12^. Moreover increasing DNMT function by methionine supplementation reduced interictal spiking in slices from epileptic rats^17^. Therefore the role of DNA methylation in seizure control is complex and may be context-dependent.

Interestingly, comparisons of our datasets with that on promoter methylation in human hippocampal TLE samples showed concordance with methylation changes^15^. For example, in the amygdala stimulation model we observe hypermethylation of LLGL2 (lethal giant larvae homolog 2). In the systemic pilocarpine we detected hypermethylation of LPHN1, (latrophilin 1) as well as hypomethylation of FER1L4 (fer-1-like 4 pseudogene) in TBI. Additionally, hypermethylation in FIGLA (folliculogenesis-specific basic helix-loop-helix) promoter was observed in the amygdala stimulation model, and previously, in patients with hippocampal sclerosis. Moreover, we found altered methylation in TTLL9 (tubulin tyrosine ligase-like family, member 9) promoter in our TBI samples, which has also been observed in patients.

The influence of altered methylation on brain function remains unclear and in the current study gene expression changes were not inversely correlated with DNA methylation changes, particularly in TBI and focal amygdala stimulation models, which is believed to contribute to gene silencing^18^. In fact, only a few genes exhibited methylation changes in gene promoter with altered in mRNA expression in the focal amygdala stimulation and systemic pilocarpine models. We identified genes showing methylation alterations in gene body including exons, introns, or on exon-intron junctions independent of gene expression. Recent studies of methylome and transcriptome data also failed to demonstrate clear effect of intragenic methylation on gene expression in different tissues, including dorsal root ganglion following peripheral nerve ligation^19^,^20^,^21^. Infact, these studies show intragenic DNA methylation plays a role in exon definition and alternative splicing, as exons with lower methylation levels tend to be excluded from mRNA while exons included or highly expressed exhibit higher levels of DNA methylation^20^,^22^,^23^. Manipulation of the exon methylation affects the inclusion of exons to the nascent mRNA^20^,^24^. The regulation of splicing mediated by intragenic methylation levels in epileptic animals remains poorly understood.

One interesting finding was the large number of differentially methylated regions located in non-genic regions. Similar observations have been made in the brains of schizophrenia or bipolar disorder subjects, as well as in experimental model of peripheral pain, indicating the role of intergenic methylation in neurological and brain disorders, however the consequences of these changes for genome functioning are not understood^21^,^25^.

Interestingly, the alterations in gene expression patterns detected here were primarily model-specific. Previously published datasets on different epilepsy models of human tissue also exhibited limited resemblance, primarily explained by differences in brain areas, the time points investigated, or the transcriptome profiling methods^26^. Our datasets describing changes in transcriptome in three different models were obtained using the same experimental procedure, which indicates that they underlie different model-specific phenomena. Previous studies have indicated downregulation of neuron specific gene expression (e.g., synaptic plasticity or learning- and memory-related genes) and upregulation of genes involved in different aspects of immune function or inflammation^26^. This is also confirmed here. However, different aspects of the immune response and inflammation predominate in each model. The functional significance of genes that are upregulated in all three models is unclear, although the importance of complement activation (Serping1), gliosis (S100a6, Msn, Gpnmb), or cell-extracellular matrix communication (Cd44) can be implicated. Transcriptome alterations support the hypothesis that different animal models have distinct characteristics with different predominating molecular features.

A surprising finding of this study was the marginal overlap of alterations in DNA methylation between experimental epilepsy models. Because we were interested in methylation patterns identifying epilepsy, we investigated models with different epileptogenic etiologies looking for changes that were not related to the model or specific laboratory conditions. Rather these changes are indicative of the epileptogenic process and possibly seizures. In the focal electrical amygdala stimulation and systemic chemoconvulsant pilocarpine models, epilepsy is sequelae of SE^27^,^28^. The two models share qualitative similarities in hippocampal pathology, including degeneration of interneurons and principal cells, gliosis, axonal sprouting, neurogenesis, and changes in extracellular matrix^29^ (see also Fig. 1). At 3 months, when tissue sampling was performed, many of the animals exhibit advanced epileptogenesis, including spontaneous seizures. The variable number of seizures, latency to first seizure and latency from last seizure to the tissue collection added to the interindividual variability and may have been a confounding factor in our molecular studies. In the TBI model, remarkable interneuron loss in the hippocampus, with milder damage to principal cells, gliosis, axonal sprouting, and neurogenesis are observed^30^(see also Fig. 1). However, according to previous studies spontaneous seizures would be expected only in approximately 15% of animals at the time point of sampling^31^,^32^. In all three models hippocampal pathology is reflected by deficits in hippocampal dependent spatial learning, which is most severe in the systemic pilocarpine model^27^,^33^,^34^. Because the structural alterations in the CA3 and dentate gyrus are more robust in systemic pilocarpine than in the focal amygdala stimulation or TBI models, at least some differences in the change in methylation status are likely due to differences in network change and phenotype. For example, differences in the level of neurodegeneration and accompanying density of glial cell population lead to imbalances between cell types, and because methylation patterns are regarded to be cell type-specific, this could explain some differences in methylation alterations between models. Additionally, spontaneous seizures being more common in focal amygdala stimulation and systemic pilocarpine models could have added to the methylation diversity because persistent epileptiform activity has been shown to influence methylation status^35^,^36^. However, methylation signatures between the two SE models were not more similar than when each of the SE models was compared with the TBI model. We hypothesize the resulting pattern of methylation is dynamic and depends on the stage of the disease development and ongoing pathology. Additionally, whether changes in methylation pattern are related to model specific changes in behavior, such as stress, mobility or disruption of circadian rhythm, cannot be excluded^37^,^38^.

Epilepsy in humans is characterized by extreme heterogeneity both in etiologies and phenotypes that is not well modeled by the current animal models and explain the poor reproducibility of preclinical data between models and difficulties in translation of potential treatment regimens to the clinical use^39^. The animal models used in this study differ in etiology and may therefore reflect features of different epilepsy syndromes in humans (post-traumatic epilepsy vs. drug refractory epilepsy). Our data indicating etiology-related differences in methylation changes in animal models are in line with human data showing pronounced differences in methylation status in epileptic hippocampus depending on the level of hippocampal sclerosis^15^. Despite the need of integrated data analysis between species, comparisons with existing human data sets are currently limited, for example, due to the use of different platform technologies (array vs. deep sequencing), limited sample numbers in existing studies, lack of human control data, or incomplete annotation of the rat genome.

In summary, we investigated alterations in genomic DNA methylation patterns which were common in three different experimental models of acquired epilepsy. Our data revealed that part of the genome-wide methylation signature distinguished injured from control animals irrespective of the initial precipitating injury, whereas other parts of the signature distinguished epileptic from control animals in a model-specific way. Our data suggest that methylation patterns depend on the epileptogenic etiology, indicating either model-specific epileptogenic mechanisms or different stages of epileptogenesis at the time of sampling. Future studies are needed to verify the specificity of our findings and identify molecular biomarkers for epileptogenesis and seizure (onset) or drug-response.

## Methods

### Epileptogenesis induced by focal electrical amygdala stimulation

Adult male Sprague-Dawley rats (Medical Research Centre, Warsaw, Poland) weighing 290–320 g were housed in a controlled environment (21–23 °C, 12 h dark/light cycles) with water and food available *ad libitum*. Starting from the day of surgery, each animal was housed in a separate cage. All animal procedures were approved by the Ethical Committee on Animal Research of the Nencki Institute and conducted in accordance with the guidelines established by the European Council Directives 2010/63/EU. The amygdala stimulation model of temporal lobe epilepsy (TLE) was performed as described by Nissinen *et al*. with modifications^27^,^40^. Briefly, surgery was performed under isoflurane anesthesia (2–2.5% in 100% O_2_) preceded by the injection of butorphanol injection (Butomidor, Richter Pharma AG, Wells, Austria; 0.5 mg/kg, i.p.). A stimulating and recording bipolar wire electrode (Plastic One Inc., Roanoke, VA, USA, # E363-3-2WT-SPC) were implanted into the left lateral nucleus of the amygdala (AP −3.6 mm, L 5.0 mm from the bregma; DV 6.5 mm from the surface of the brain)^41^. A stainless steel screw electrode (Plastic One Inc., Roanoke, VA, USA, #E363/20) was implanted contralaterally into the skull over the right frontal cortex (AP + 3.0 mm, L 2.0 mm from the bregma) as a surface EEG recording electrode. Two stainless steel screw electrodes were placed bilaterally over the cerebellum (AP − 10.0 mm, L 2.0 mm from the bregma) as ground and reference electrodes. The socket contacts of all electrodes were placed in a multi-channel electrode pedestal (Plastic One Inc., Roanoke, VA, USA, #MS363) attached to the skull with dental acrylate (Duracryl Plus). After 2 weeks of recovery, animals were electrically stimulated via the intra-amygdala electrode to evoke SE. Stimulation consisted of a 100-ms train of 1-ms biphasic square-wave pulses (400 μA peak to peak) delivered at 60 Hz every 0.5 s for 30 min. If the animal did not enter SE, stimulation was continued for an additional 10 min. The SE was stopped 1.5–2 h after stimulation via an intraperitoneal injection of diazepam (20 mg/kg; Relanium, Polfa, Warsaw, 5 mg/ml). If the first dose of diazepam did not suppress SE, the animal received subsequent doses of diazepam (5 mg/kg). Age-matched control animals had electrodes implanted but did not receive electrical stimulation.

Stimulated rats (n = 5) were monitored with video EEG (Comet EEG, Grass Technologies, West Warwick, RI) for 2 weeks prior the end of the experiment to determine the presence of spontaneous seizures. Spontaneous seizures were identified from EEG recordings by browsing the EEG manually on the computer screen. An electrographic seizure was defined as a high frequency (>8 Hz), high amplitude (>2 × baseline) discharge lasting for at least 5 s. All stimulated animals enrolled had spontaneous seizures. The frequency of seizures was 3.9 ± 2.7 seizures/day. Time-matched control sham operated animals had electrodes implanted but were not stimulated (n = 5).

### Epileptogenesis induced by TBI

Adult male Sprague-Dawley rats (Harlan, Netherlands) weighing 330–371 g at the time of TBI were housed in a controlled environment (21–23 °C, 12 h dark/light cycle, 50–60% relative humidity) with water and food available *ad libitum*. All animal procedures were approved by the Committee for the Welfare of Laboratory Animals of the University of Eastern Finland in accordance with the guidelines established by the European Community Council Directives 2010/63/UE. The induction of TBI was performed according the lateral fluid percussion (LFP) method described by McIntosh *et al*.^42^ and Kharatishvili *et al*.^31^. Animals (n = 18) were anesthetized with an intraperitoneal (i.p.) injection of a solution (6 ml/kg) containing sodium pentobarbital (58 mg/kg), chloral hydrate (60 mg/kg), magnesium sulphate (127.2 mg/kg), propylene glycol (42.8%) and absolute ethanol (11.6%) and placed in a Kopf stereotactic frame (David Kopf Instruments, Tujunga, CA, USA). First, the skull was exposed with a midline skin incision followed by extraction of the periosteum. The left temporal muscle was detached from the lateral ridge and then, a circular craniectomy (Ø 5 mm) was performed over the left parietal lobe midway between the lambda and bregma keeping the dura mater intact. The craniectomy edges were sealed with a modified Luer-Lock cap that was filled with saline, and the calvaria was covered with dental acrylate (Selectaplus CN, Dentsply DeTrey GmbH, Dreieich, Germany). Lateral FPI was induced 90 min after the administration of anesthesia by connecting the rat to a fluid-percussion device (AmScien Instruments, Richmond, VA, USA) via a female Luer-Lock fitting. The mean severity of the impact was 3.32 ± 0.01 atm. Control animals (n = 12) received anesthesia and underwent all surgical procedures but without lateral FPI. Mortality within 48 h post-TBI was 17% (3 of 18 rats). For further analysis, we included 5 TBI and 5 control rats selected from animals that survived until end of the follow-up period. TBI animals were not subjected to EEG-monitoring. They did not express handling-related seizures. Previous data indicate that at this time point no more that 15% rats have epilepsy^31^.

### Epileptogenesis induced by systemic pilocarpine

The animal handling procedure and first analysis of methylome and transcriptome data obtained from this cohort of rats were previously published by Kobow *et al*.^10^. Here we reanalyzed and extended the analysis and present new findings when relevant.

Animal procedures have been described elsewhere^10^. In short, the experimental study design was approved by the local animal care and use committee in accordance with the European Communities Council Directive (54-2532.1-23/09, Directive 2010/63/EU). Male Wistar rats weighting 300–350 g (Charles-River, Germany; n = 14) were kept in individual cages under controlled environmental conditions (12 h dark/light cycles, 20–23 °C and 50% relative humidity) with drinking and feeding ad libitum. Representative animals were assigned to continuous video-electroencephalography monitoring (vEEG; DSI, St. Paul, MN, USA). Electrodes were implanted before seizure induction. Rats were deeply anesthetized with an intraperitoneal injection of a ketamine (57 mg/kg) and xylazine (9 mg/kg) mixture and placed in a stereotaxic apparatus (Bilaney Consultants, Düsseldorf, Germany). Two epidural EEG electrodes, i.e. touching but not penetrating the dura, were implanted (2 mm lateral to the sagittal suture and 5 mm anterior to the lambda suture, 1 mm diameter) The transmitter (F40-EET, DSI, St. Paul, MN, USA) was placed into a subcutaneous pocket along the animal’s dorsal flank. Implanted animals had time to recover for 1 week before further procedures. There were no differences in baseline EEG between animals before further treatment.

To induce status epilepticus (SE), animals were injected with a single high dose of the muscarinic receptor agonist pilocarpine (PILO; 340 mg/kg, i.p.; Sigma-Aldrich, Steinheim, Germany). Peripheral cholinergic effects were minimized by administration of methyl scopolamine (1 mg/kg, s.c.; 30 min before injection of pilocarpine; TCI Europe NV, Zwijndrecht, Belgium). Animals that experienced no SE within 45 min after first pilocarpine administration were treated for a second time with half the application dosis (175 mg/kg, i.p.). After 60 min SE duration, animals received administration of diazepam (8 mg/kg, i.m.; Sigma-Aldrich). One hour following diazepam treatment glucose depots (2 × 5 ml) were subcutaneously injected to help animals to recover. Age matched control rats (CTRL; n = 5) received methyl scopolamine and saline (0.9% NaCl; Sigma-Aldrich) injections only.

Implanted animals were video-EEG monitored 24/7 starting from day of implantation until 12 weeks after initial precipitating injury. Behavioral seizures were quantified. The average time to first seizure was 8,4 ± 1,8 days post-SE. Seizure frequency per week was 24.5 ± 4.7 seizures/week.

### Tissue collection

Tissue was collected at 3 months after SE or TBI induction. For tissue collection, the rats were anesthetized with CO_2_ (amygdala stimulation model), 4% isoflurane (TBI), or ether (pilocarpine model), and decapitated with a guillotine. The left hippocampus was rapidly isolated, and tissue containing CA3 and dentate gyrus were excised on a platform placed on ice. For downstream applications, the CA3/dentate gyrus was homogenized in ice cold 1 × PBS and divided into equal volumes for DNA and RNA extraction exactly as described by Kobow *et al*.^10^. Samples from each animal were processed individually. Samples were stored at −80 °C until use.

### DNA methylation profiling

The same procedure was used for all three models. DNA was extracted from a tissue piece containing the CA3 and dentate gyrus using the DNeasy Blood and Tissue Kit (Qiagen, Hilden, Germany) according to the manufacturer’s instructions. Massive parallel sequencing of enriched methylated DNA was performed as described previously^43^. Briefly hippocampal DNA from each rat (amygdala stimulation: control n = 4, SE n = 4; pilocarpine: control n = 5, SE n = 4; TBI: control n = 4, TBI n = 5) was fragmented to a median size of 200–300 bp and subjected to methylated DNA capture according to the MethylMiner protocol (Invitrogen, Darmstadt, Germany), exclusively enabling capture of methylated double-stranded DNA. Fragmented and enriched DNA was eluted at high salt concentrations (2 M NaCl). Five nanograms of enriched DNA for amygdala stimulation, 10 ng for pilocarpine, and 5 ng for TBI was used in library preparation using the NEBNext DNA Library Prep Reagent Set for Illumina (New England Biolabs, Frankfurt/Main, Germany). The quality of sequencing libraries was assayed using the Shimadzu MultiNA capillary electrophoresis system (Shimadzu, Kyoto, Japan). Libraries were sequenced at a concentration of 10 pM using the Illumina Genome Analyzer IIx (Illumina, San Diego, CA, USA) with a 36 bp single read length. Image analysis and base-calling were performed using the OLBv1.8 software. Sequenced tags were aligned to the rat genome RN4 using BWA (version 0.5.9) with default parameter^44^.

Profiles of DNA methylation were compared between all pairwise combinations of samples for each model separately using the MACS peak calling software (version 1.4.0 rc2) with fixed shift size of 75 bp and significance cut-off of 10E-05^45^. Genomic regions showing different methylation patterns between pairs of samples were merged using BEDTools^46^. Duplicate reads that aligned to the same location in a given sample were removed from further analysis. The number of read tags aligning to each region was summarized using a custom python script producing a matrix of counts (tags per region per sample). The regions were non-differentially filtered for regions in which the sum of tag counts was below the 50th percentile. These count data were tested for differential tag abundance using Bayesian shrinkage of negative binomial model implemented in edgeR^47^, and normalized using trimmed mean normalization^48^. Circos plot (Fig. 2e) was used to visualize methylation events (cut-off p < 0.01) on the rat genome ideogram^49^. First analyses of alterations in DNA methylation in systemic pilocarpine model have been previously published by Kobow *et al*.^10^.

Heatmaps were generated in the R environment with the gplots package^50^,^51^. Unsupervised clustering was performed by taking the trimmed mean normalized values for differential regions as defined by edgeR analysis (cut-off p < 0.01). These values were normalized to the standard normal distribution. Methylation events and samples were ordered with clustering complete-linkage method together with a Pearson correlation based distance measure (Fig. 2a–c).

### Establishing common differentially methylated regions in the different TLE models

To detect of common features in methylation for at least two models, regions with increased or decreased methylation in each model (cut-off p < 0.01) were used. To identify common differentially methylated regions between models, we used non-adjusted p-values to include regions with low significance; however occurrence in at least two models was required. Regions, in which alterations in methylation status were common for two or three models were detected via intersections with BEDTools v2.16.1^46^. Overlapping regions (≥1bp) were considered common when the level of methylation between epileptic and control animals changed in the same direction. Circos plot (Fig. 2f) was used to map common methylation events to the rat genome ideogram^49^. Heatmap presenting overlapping regions with consistent change (cut-off p < 0.01) in DNA methylation in at least two models of epilepsy (Fig. 2d) was generated in the R environment with the gplots package^50^,^51^. Unsupervised clustering was performed by taking the trimmed mean normalized values for differential regions as defined by edgeR analysis for each model of epilepsy separately. These values were normalized to the standard normal distribution for each model separately. Methylation events and samples were ordered with clustering complete-linkage method together with Pearson correlation based distance measure.

### Distribution of differentially methylated regions across genomic features

Information about genomic features: CpG islands, SNPs, promoters (200 bp or 5 kb upstream of transcription start), TSSs, gene bodies, 5′UTRs, coding exons, introns, 3′UTRs, and non-genic regions was obtained for rat genome version RN4 from Ensembl via the UCSC Genome Table Browser^52^. We intersected genomic features with differentially methylated regions of DNA between epileptic and control animals (cut-off p < 0.01) (overlap 1 bp or more) with BEDTools 2.16.1^46^ and compared the distribution of DNA methylation changes for three models for all of these genomic features. Differentially methylated regions were normalized and expressed as log2 odds ratio compared with non-differentially methylated genomic features separately for hyper- and hypomethylation events. Barplots in Fig. 2h–j present the mean frequency of observed methylation changes with upper and lower 95% confidence intervals for the genomic features. We also compared hyper- and hypomethylation events at specific genomic features for each model. Barplot in Fig. 1k presents log2 odds ratio of the number of increased *versus* decreased methylation regions across genomic features with upper and lower 95% confidence intervals. Significance was estimated with two-sided Fisher’s exact test and p < 0.05 was considered significant. Barplots were created, and statistical calculations were performed in the R environment version 3.0.2^50^.

### Gene expression profiling

The mRNA expression profiling procedure was the same for all three models. The numbers of samples analyzed was as follows: amygdala stimulation - control n = 5, SE n = 4; pilocarpine - control n = 5, SE n = 5; TBI - control n = 3, TBI n = 5. Total RNA was extracted using the Trizol method, followed by DNAse digestion. RNA quality was verified with the Shimadzu MultiNA capillary electrophoresis system (Shimadzu). Following Dynabead Oligo(dT) enrichment (Invitrogen), mRNA was prepared into sequence-ready libraries with the NEBNext mRNA Library Prep Reagent Set for Illumina (New England Biolabs). Libraries were sequenced at a concentration of 10 pM (pilocarpine) or 13 pM (amygdala stimulation and TBI) on the Illumina Genome Analyzer IIx (Illumina, San Diego, CA, USA) with a 36-bp single read length. Image analysis and base-calling were performed as described above. Sequenced tags were aligned to the rat genome RN4 using BWA (version 0.5.9) with default alignment parameters^44^. The numbers of read tags aligning to each gene were extracted using a custom python script that produces a matrix of counts with regions based on the Ensembl transcript annotation (version 66). Genes were non-differentially filtered for tag counts sums below the 30th percentile. These count data were tested for differential tag abundance using Bayesian shrinkage of a negative binomial model implemented in edgeR^47^, and normalized using trimmed mean normalization^48^.

Heatmaps for individual models were generated in the R environment with gplots package^50^,^51^. Unsupervised clustering was performed by taking the trimmed mean normalized values for differential genes as defined by edgeR analysis (cut-off p < 0.001). These values were normalized to the standard normal distribution. Genes and samples were ordered with clustering complete-linkage method together with a Pearson correlation based distance measure (Fig. 3a–c) and normalized values. For heatmaps presenting unsupervised clustering of all samples and models, z-scores were calculated for each model separately and a Pearson correlation-based distance measure was used.

For the seven genes upregulated in all three models of epilepsy we analyzed direct and indirect experimentally confirmed interactions between genes and gene products using IPA (Ingenuity Pathway Analysis, Quiagen, Redwood City, CA) (Fig. 3g).

### Comparison of functional enrichment of GO terms for the Biological Process branch and biological pathways from KEGG and Reactome

Analysis of enriched GO terms from the Biological Process branch was performed separately with the gProfiler webservice^53^,^54^ via the GOsummaries R package^55^ for upregulated and downregulated genes for each epilepsy model (Fig. 3h). Whole genome was used as a reference set. The function ‘gosummaries’ from the GOsummaries package was used with default arguments using human GO, KEGG and Reactome database as targets.

### Gene set enrichment expression analysis

A gene set enrichment analysis (GSEA) was performed separately for each model using the ranked mRNA results and sets of differentially methylated genes (cut-off p < 0.001)^56^. Rank scores for differential mRNA expression were calculated as -log10(p-value) multiplied by the sign of the edgeR fold change such that upregulated genes had positive scores and downregulated genes had negative scores. Scores for genes that were represented by more than one transcript were calculated with the lowest *p*-value. The obtained rank scores were used to test for relationships between gene expression and DNA methylation using the GSEA preranked method based on 1000 gene set permutations. Sets of differentially methylated genes were derived by filtering the differentially methylated regions between epileptic and control animals (p < 0.001 as determined by edgeR analysis) and separated into increased and decreased methylation. Promoters were defined as regions 5 kb upstream of TSS. An initial analysis of alterations in transcriptome from animals with pilocarpine induced epilepsy was previously published by Kobow *et al*.^10^.

### Differentially expressed genes in which methylation changes occurred

Lists of differentially expressed genes (cut-off p < 0.001) were obtained separately for each model of epilepsy. Additionally, a list of differentially methylated genes (cut-off p < 0.001), in which methylation changes occurred in gene bodies or promoters defined as 5 kb upstream from TSS was obtained. Lists of differentially expressed and methylated genes were intersected for each model separately to obtain list of relationships between methylation and expression for each animal model presented (Fig. 4d).

## Additional Information

**Accession codes**: Methyl-Seq and mRNA-Seq data for the focal amygdala stimulation model were deposited in the NCBI Gene Expression Omnibus under access id GSE75403, the systemic pilocarpine model with access id GSE50080, and the TBI model under access id GSE75121.

**How to cite this article**: Dębski, K. J. *et al*. Etiology matters – Genomic DNA Methylation Patterns in Three Rat Models of Acquired Epilepsy. *Sci. Rep.* **6**, 25668; doi: 10.1038/srep25668 (2016).

## Supplementary Material

Supplementary Information
