# Supplementary material for: Etiology matters – Genomic DNA Methylation Patterns in Three Rat Models of Acquired Epilepsy
Source: Sci Rep. 2016 May 9;6:25668. doi: 10.1038/srep25668 (PMC4860710; doi:10.1038/srep25668)
Supplement: Supplementary Information [file srep25668-s1.pdf]

Etiology matters – Genomic DNA Methylation Patterns in Three Rat Models of Acquired Epilepsy

K. Dębski, A. Pitkanen, N. Puhakka, A. M. Bot, I. Khurana, H. KN, M. Ziemann, A. Kaspi, A. El-Osta, K. Lukasiuk, K. Kobow

Supplementary Table 1: List of genes with altered expression levels in the amygdala stimulation model of epilepsy

Name	logFC	directedFC	PValue	adj.p.value	Gene_id	Direction
NM_022197	3,71669488	13,14730214	7,65369E-10	1,50464E-05	Fos	upregulated
NM_017128	4,473947993	22,22248106	8,29568E-09	4,94326E-05	Inhba	upregulated
NM_031055	4,604087747	24,32027686	1,0058E-08	4,94326E-05	Mmp9	upregulated
NM_001025650	1,008612193	2,011974741	0,000000024	9,43091E-05	Dusp11	upregulated
NM_012614	3,470245844	11,08276416	4,08634E-08	0,000130954	Npy	upregulated
NM_001100474	2,793564777	6,933408565	0,000000062	0,000152271	Ccnf	upregulated
NM_021586	2,410499259	5,316582793	7,574E-08	0,000160459	Ltbp2	upregulated
NM_001107052	2,291819659	4,896733427	8,16209E-08	0,000160459	Arl4d	upregulated
NM_001034199	3,082090548	8,468406647	1,17123E-07	0,000209319	Nptx2	upregulated
NM_001079890	3,897185248	14,89943014	1,36691E-07	0,000223935	Gprc5a	upregulated
NM_053727	1,748338445	3,359714042	1,70236E-07	0,000227886	Nfil3	upregulated
NM_013046	7,534337408	185,3794363	1,73879E-07	0,000227886	Trh	upregulated
NM_053633	3,554509474	11,74935353	0,000000275	0,000337891	Egr2	upregulated
NM_017232	2,768677919	6,814831182	4,42472E-07	0,000457819	Ptgs2	upregulated
NM_013151	1,266750314	2,406189579	4,80139E-07	0,000471953	Plat	upregulated
NM_053572	-1,991505373	-3,976517095	5,39405E-07	0,000504961	Pcdh21	downregulated
NM_001031656	1,878391635	3,676649465	6,18427E-07	0,000545887	Serinc2	upregulated
NM_139090	1,761873691	3,391382927	7,68621E-07	0,000629596	Acvr1c	upregulated
NM_030997	1,852322557	3,610810122	8,97373E-07	0,000705659	Vgf	upregulated
NM_012795	2,636906853	6,219966686	1,0931E-06	0,000795895	Gp5	upregulated
NM_019137	2,218635579	4,654530266	1,46857E-06	0,000962356	Egr4	upregulated
NM_001130564	1,059677472	2,084465468	1,60891E-06	0,001020307	Efr3a	upregulated
NM_001025680	1,412346325	2,661696957	1,90765E-06	0,001133434	Gpr4	upregulated
NM_080782	2,337247667	5,053376486	1,96026E-06	0,001133434	Cdkn1a	upregulated
NM_012551	1,367814721	2,580793519	2,89325E-06	0,001458422	Egr1	upregulated
NM_053453	1,280331399	2,428947654	4,05053E-06	0,001895938	Rgs2	upregulated
NM_022187	2,307582129	4,950527056	4,84321E-06	0,002163924	Plk3	upregulated
NM_019361	2,886297515	7,393705165	5,34868E-06	0,002298315	Arc	upregulated
NM_031135	2,092022303	4,263452854	0,00000554	0,002317232	Klf10	upregulated
NM_001011936	2,327889552	5,020703583	5,82189E-06	0,002384429	Bag3	upregulated
NM_031821	1,468415143	2,767177414	6,03233E-06	0,002420194	Plk2	upregulated
NM_021584	1,780083244	3,43445991	6,26811E-06	0,002464494	Dclk1	upregulated
NM_001108593	0,576753976	1,49148966	7,1389E-06	0,002647995	Sec23b	upregulated
NM_001107082	0,633835203	1,551684452	7,95756E-06	0,002896993	Etv5	upregulated
NM_001109102	3,007223549	8,040156308	9,17416E-06	0,003279179	Fam167a	upregulated
NM_001106684	1,787634826	3,452484233	9,5567E-06	0,003354914	Rhbdl2	upregulated
NM_012959	1,041642392	2,058569836	1,31727E-05	0,004389175	Gfra1	upregulated

NM_053455	1,840591703	3,581568916	1,37617E-05	0,004509007	Fgl2	upregulated
NM_134454	3,775063581	13,69012373	1,43983E-05	0,00464027	Angpt2	upregulated
NM_030863	1,513676377	2,855367376	1,47382E-05	0,004673202	Msn	upregulated
NM_001106081	1,343932053	2,538422216	1,56259E-05	0,00479984	Tll1	upregulated
NM_001130573	3,993811103	15,93150994	1,68235E-05	0,005084422	Maff	upregulated
NM_013026	2,987913461	7,933257961	1,73343E-05	0,005084422	Sdc1	upregulated
NM_053819	3,418400106	10,69155733	1,75869E-05	0,005084422	Timp1	upregulated
NM_057130	-1,054215761	-2,07658908	1,88343E-05	0,005289465	Hrk	downregulated
NM_031591	0,910630068	1,879866313	2,08063E-05	0,005680992	Pecam1	upregulated
NM_133551	2,263406401	4,801237827	2,20209E-05	0,005930258	Pla2g4a	upregulated
NM_212507	-1,581030617	-2,991835009	2,57695E-05	0,006754697	Ltb	downregulated
NM_212509	-0,664519616	-1,585040403	2,64205E-05	0,006834211	Nfkbil1	downregulated
NM_001011947	1,10776211	2,155110899	2,69002E-05	0,006867943	Rai14	upregulated
NM_001134884	1,880465984	3,681939659	2,99472E-05	0,00745231	Scube1	upregulated
NM_001169127	0,759885997	1,693356809	3,14955E-05	0,007739631	Tspan14	upregulated
NM_001110335	1,430967782	2,696275249	3,19816E-05	0,00775901	Vegfa	upregulated
NM_001108278	-0,562300381	-1,476621819	3,23637E-05	0,00775901	Sat2	downregulated
NM_001113522	0,776748581	1,713265312	3,30224E-05	0,007819924	Arhgef7	upregulated
NM_017034	2,882938814	7,376512092	3,34134E-05	0,007819924	Pim1	upregulated
NM_001191577	1,284304947	2,435646813	0,000034323	0,007938296	Midn	upregulated
NM_022294	0,918833733	1,890586335	3,66872E-05	0,008071006	Eltd1	upregulated
NM_023985	2,737087384	6,667229478	0,000037133	0,008071006	Trib1	upregulated
NM_021836	0,997656504	1,996753862	3,73601E-05	0,008071006	Junb	upregulated
NM_031970	4,262912409	19,19837648	3,77975E-05	0,008076596	Hspb1	upregulated
NM_013040	0,717269772	1,644067773	3,84867E-05	0,008076596	LOC100360403	upregulated
NM_001137642	3,635496443	12,42777776	3,86184E-05	0,008076596	Kif18a	upregulated
NM_001025670	1,787219249	3,451489868	3,90527E-05	0,008081446	Agpat9	upregulated
NM_053349	3,326859857	10,03424286	4,33554E-05	0,008878374	Sox11	upregulated
NM_017214	1,714975969	3,28291175	4,46242E-05	0,008896569	Rgs4	upregulated
NM_133298	1,302707918	2,466914848	4,59742E-05	0,008896569	Gpnmb	upregulated
NM_001013191	1,279323958	2,4272521	0,000046138	0,008896569	Cbfb	upregulated
NM_001109422	3,827092449	14,19285034	4,65843E-05	0,008896569	Bcl3	upregulated
NM_012543	-0,941958661	-1,921134679	0,000047427	0,008896569	Dbp	downregulated
NM_024127	2,285048968	4,873806478	4,76196E-05	0,008896569	Gadd45a	upregulated
NM_001008321	2,14001544	4,407667635	0,00004788	0,008896569	Gadd45b	upregulated
NM_001134599	1,076721588	2,109237553	4,79697E-05	0,008896569	Flna	upregulated
NM_013064	2,926011332	7,600062759	4,85896E-05	0,008927323	Hcrtr1	upregulated
NM_001108393	0,95769723	1,942207348	4,99906E-05	0,009016187	Zmiz1	upregulated
NM_001108940	-0,723140371	-1,650771426	5,07152E-05	0,009063724	Bhlhe22	downregulated
NM_019143	1,008058428	2,011202612	5,29354E-05	0,009291587	Fn1	upregulated
NM_001134856	0,777739031	1,71444192	5,39912E-05	0,009393033	Cdc14a	upregulated
NM_021859	-0,402710431	-1,321989237	5,59141E-05	0,009642244	Matk	downregulated
NM_019162	3,785874882	13,79310046	5,87775E-05	0,00996126	Tac2	upregulated
NM_001015017	1,257714686	2,391166659	6,07421E-05	0,010206233	Olfm2	upregulated
NM_031327	4,189094788	18,24077077	6,18085E-05	0,010210869	Cyr61	upregulated
NM_031528	1,383313491	2,60866826	6,32689E-05	0,010365032	Rara	upregulated

NM_001105751	0,477339568	1,39217403	6,47957E-05	0,010527427	F1M6K7_RAT	upregulated
NM_012912	3,493249888	11,26089733	6,94398E-05	0,011098508	Atf3	upregulated
NM_001034128	0,836634156	1,785878777	7,32052E-05	0,01151313	Pald	upregulated
NM_019328	1,042972108	2,060468071	0,000074488	0,011598219	Nr4a2	upregulated
NM_021693	2,455422853	5,484738558	7,49262E-05	0,011598219	Sik1	upregulated
NM_019905	1,532900698	2,893670591	7,58679E-05	0,011652238	Anxa2	upregulated
NM_199397	1,058453501	2,082697774	0,000079282	0,011912188	Panx1	upregulated
NM_001006958	0,673506245	1,594944524	7,93782E-05	0,011912188	Sdad1	upregulated
NM_001108227	1,741083748	3,342861881	8,41474E-05	0,012532231	Wnt10a	upregulated
NM_001107113	1,271884858	2,414768451	8,57599E-05	0,012629857	Bach1	upregulated
NM_001037179	0,959156201	1,94417246	8,66626E-05	0,012629857	Lrrc8c	upregulated
NM_001002851	-0,579207078	-1,494027888	8,91972E-05	0,012826052	Nenf	downregulated
NM_001007641	0,734859518	1,664235407	8,93824E-05	0,012826052	Rnd3	upregulated
NM_001109536	3,263203972	9,601128392	9,26107E-05	0,013192993	Ptx3	upregulated
NM_178104	0,844961617	1,796216957	9,66626E-05	0,013573506	Cpg1	upregulated
NM_001105716	1,935451928	3,824979267	9,75892E-05	0,013606428	Crabp1	upregulated
NM_138894	1,739227956	3,338564599	0,000104813	0,014309144	Grasp	upregulated
NM_021597	1,192926294	2,28615987	0,000106654	0,014360971	Eif2c2	upregulated
NM_024486	0,974794113	1,965360701	0,000108455	0,014406146	Acvr1	upregulated
NM_001085353	0,713087316	1,639308428	0,000110235	0,014544384	WLS_RAT	upregulated
NM_012945	2,800548178	6,967051259	0,000116799	0,015106276	Hbegf	upregulated
NM_001004090	0,784924009	1,723001561	0,000118095	0,01516392	Tspan5	upregulated
NM_001109247	1,260671881	2,396073031	0,000118788	0,01516392	Arhgap25	upregulated
NM_001134597	0,592210621	1,507554983	0,0001226	0,015549648	Fam135a	upregulated
NM_178094	1,428467424	2,691606339	0,000123423	0,015553701	Itpkc	upregulated
NM_199408	0,825849567	1,772578557	0,000131829	0,016507179	WLS_RAT	upregulated
NM_001100900	0,570175882	1,484704563	0,000133967	0,016668648	Ss18	upregulated
NM_001107834	1,097241269	2,139451937	0,000137063	0,016946697	Pbx3	upregulated
NM_001008320	1,014052996	2,01957678	0,000138796	0,017053672	Rhoj	upregulated
NM_001105769	0,491214235	1,405627415	0,000139731	0,017061937	Tbc1d24	upregulated
NM_019274	0,806965835	1,749528099	0,000142852	0,017335397	COLQ_RAT	upregulated
NM_031514	1,039943982	2,056147814	0,000151599	0,0181725	Jak2	upregulated
NM_138827	0,860034502	1,815081718	0,000158203	0,018759702	Slc2a1	upregulated
NM_001011922	0,864897146	1,821209823	0,000158406	0,018759702	Nedd9	upregulated
NM_001003403	2,261426147	4,794652132	0,000160718	0,018919467	Apold1	upregulated
NM_134382	0,401087316	1,320502759	0,000165241	0,019221703	Elovl5	upregulated
NM_012924	2,517465483	5,725753187	0,00016764	0,019338361	Cd44	upregulated
NM_001134559	0,799862633	1,740935354	0,000168774	0,019338361	RGD1308907	upregulated
NM_001107807	1,223375786	2,334924314	0,00017542	0,019902156	Pmepa1	upregulated
NM_001113521	0,733903473	1,663132917	0,000176152	0,019902156	Arhgef7	upregulated
NM_019372	1,716107901	3,28548852	0,000181462	0,020336015	Pdp1	upregulated
NM_199093	1,524405387	2,87668128	0,00018413	0,020336015	Serping1	upregulated
NM_019346	0,903805273	1,870994443	0,000187356	0,020563961	Slc14a1	upregulated
NM_001108004	1,306830824	2,473974833	0,00018928	0,020563961	Dnajb5	upregulated
NM_031548	2,040170846	4,112942337	0,000194166	0,020886588	Scnn1a	upregulated
NM_001110333	1,374295067	2,592412089	0,000194427	0,020886588	Vegfa	upregulated

NM_001103352	-0,52205741	-1,436001653	0,0001987	0,021229596	Kifc3	downregulated
NM_001107197	1,516826875	2,861609622	0,000200334	0,021288428	Igsf9	upregulated
NM_012825	0,520143037	1,434097425	0,000202767	0,021431122	Aqp4	upregulated
NM_134350	-0,712547272	-1,6386949	0,000204113	0,021434194	Mx2	downregulated
NM_001108733	1,161034146	2,236176627	0,000204976	0,021434194	Dot1l	upregulated
NM_001009704	-0,669964932	-1,591034293	0,000210207	0,021749812	Sipa1l2	downregulated
NM_030839	-0,424936753	-1,342513647	0,000211825	0,021802426	Syt13	downregulated
NM_001003711	-0,674449306	-1,595987449	0,000216588	0,022176526	Jph4	downregulated
NM_031834	-0,693651654	-1,617372127	0,000221286	0,02231159	Sult1a1	downregulated
NM_012488	0,789826668	1,728866736	0,000230416	0,023110923	A2m	upregulated
NM_001034006	1,09655059	2,138427937	0,000236707	0,023513066	Acap2	upregulated
NM_001129880	0,911730862	1,881301222	0,000238013	0,023513066	LOC683626	upregulated
NM_001007144	1,241367095	2,364224596	0,000243215	0,023670089	Adfp	upregulated
NM_031836	1,290396574	2,445952816	0,000247487	0,023967263	Vegfa	upregulated
NM_001025051	2,074712305	4,212604004	0,000258647	0,024546887	Fam110c	upregulated
NM_052801	0,665548938	1,586171688	0,000259187	0,024546887	Vhl	upregulated
NM_001009405	0,898774572	1,864481616	0,000260349	0,024546887	Arhgap29	upregulated
NM_001107253	0,532122105	1,446054679	0,000262221	0,024546887	Kctd6	upregulated
NM_145721	-0,44414302	-1,360505718	0,000263848	0,024546887	Cdk5rap1	downregulated
NM_012853	1,052836355	2,074604537	0,000264542	0,024546887	Htr4	upregulated
NM_022199	1,795913694	3,472353179	0,000265039	0,024546887	Dusp4	upregulated
NM_001107204	1,125782515	2,182198764	0,000267208	0,024546887	Lrrc8b	upregulated
NM_001107979	1,537243946	2,902395141	0,000269324	0,024548875	Fhl3	upregulated
NM_133306	1,720518502	3,29554827	0,000269939	0,024548875	Olr1	upregulated
NM_053794	0,411821772	1,33036468	0,000270975	0,024548875	WNK1_RAT	upregulated
NM_012643	1,304075429	2,469254312	0,000274157	0,024723193	F1MAG5_RAT	upregulated
NM_012892	-0,642617578	-1,561159106	0,000275924	0,024768926	Accn1	downregulated
NM_001107272	-0,535066064	-1,449008506	0,000277463	0,02479383	Pnma2	downregulated
NM_001106132	3,361311605	10,27674589	0,000280137	0,02491953	Loxhd1	upregulated
NM_001109571	-0,563254964	-1,477599173	0,000281442	0,024922826	Polr3gl	downregulated
NM_001001504	-0,7588936	-1,692192388	0,00028627	0,025124004	F1M710_RAT	downregulated
NM_001009692	-0,334235915	-1,260709531	0,000289463	0,025291348	Sh3glb2	downregulated
NM_001108834	0,842548463	1,793214988	0,00029715	0,025848095	Stac2	upregulated
NM_001100722	1,240566887	2,362913614	0,000323589	0,027602868	Lingo1	upregulated
NM_001107335	-0,515295014	-1,429286382	0,00032423	0,027602868	Dapk1	downregulated
NM_022259	0,761708313	1,695497095	0,000324343	0,027602868	Cd244	upregulated
NM_203338	0,673979746	1,59546808	0,000329026	0,027761267	Vkorc1l1	upregulated
NM_001031813	-0,956264616	-1,94027967	0,000329296	0,027761267	ARHL1_RAT	downregulated
NM_001108601	-0,486348366	-1,400894552	0,000330441	0,027761267	Dhx35	downregulated
NM_013000	0,589933244	1,505177098	0,000338851	0,028346713	Pam	upregulated
NM_021688	0,92792866	1,902542471	0,000343106	0,02858103	Kcnk1	upregulated
NM_001013127	1,771786121	3,414764583	0,000346181	0,028664319	Tagln2	upregulated
NM_013047	-1,005689346	-2,007902681	0,000347022	0,028664319	Trhr	downregulated
NM_001107352	0,882518364	1,84359066	0,000354593	0,029004752	Hist1h2bh	upregulated
NM_001098724	0,975745177	1,966656747	0,000356238	0,029004752	Necab3	upregulated
NM_024400	3,237505823	9,431621502	0,000356376	0,029004752	Adamts1	upregulated

NM_013044	0,613575228	1,530046213	0,000357045	0,029004752	Tmod1	upregulated
NM_001105857	0,712657391	1,638819985	0,000360503	0,029165107	Pycr1	upregulated
NM_001033066	1,002621222	2,003637088	0,000362436	0,029201337	Ddhd1	upregulated
NM_001012072	0,582980131	1,497940304	0,00036737	0,029321763	Ppp1r3c	upregulated
NM_001014127	-0,424847166	-1,342430283	0,000376648	0,029618102	Snrnp35	downregulated
NM_130420	0,746105611	1,677259146	0,000379694	0,029738681	Trim9	upregulated
NM_013134	0,72352497	1,651211553	0,0003856	0,030081391	Hmgcr	upregulated
NM_017325	1,968411354	3,913369548	0,000393521	0,030510482	Runx1	upregulated
NM_031040	0,368735303	1,291220424	0,000395584	0,030510482	Grm7	upregulated
NM_001017487	0,522192966	1,436136587	0,000396334	0,030510482	Bri3bp	upregulated
NM_053490	0,408506474	1,327311026	0,000397612	0,030510482	Xpo1	upregulated
NM_001108052	0,840608712	1,790805572	0,00039886	0,030510482	Rin3	upregulated
NM_001191763	-1,008684278	-2,012075273	0,000406619	0,030983451	Mycl1	downregulated
NM_001047102	0,520106208	1,434060816	0,000414309	0,031206521	Cadm2	upregulated
NM_001108934	-0,693197462	-1,616863023	0,000418162	0,031222978	Mblac2	downregulated
NM_001012176	-0,891580676	-1,855207652	0,000419739	0,031222978	Rsph1	downregulated
NM_012771	0,957939998	1,942534199	0,000427442	0,031590518	Lyz2	upregulated
NM_013083	0,374911478	1,296759985	0,000436091	0,031789484	Hspa5	upregulated
NM_001011997	0,686018307	1,608837157	0,000439784	0,031789484	Tmod3	upregulated
NM_022685	0,978952985	1,97103444	0,000439836	0,031789484	Rem2	upregulated
NM_012620	2,565562338	5,919857046	0,000446452	0,032044216	Serpine1	upregulated
NM_001005384	2,524238341	5,752696427	0,000449415	0,03212747	Osmr	upregulated
NM_001107242	0,404151889	1,323310752	0,000453966	0,032335198	Pex13	upregulated
NM_001024897	1,152270701	2,222634463	0,00045711	0,032424394	Ehd2	upregulated
NM_001191883	-0,348763251	-1,27346848	0,000458962	0,032424394	Pcbp4	downregulated
NM_053326	0,64458559	1,563290171	0,000460166	0,032424394	Pdlim5	upregulated
NM_031530	6,409403507	85,00074065	0,000463025	0,032509327	Ccl2	upregulated
NM_031531	7,072236015	134,5721456	0,000470057	0,032885608	Serpina3n	upregulated
NM_017086	2,086028507	4,24577673	0,00047293	0,032969271	Egr3	upregulated
NM_031653	1,244111076	2,368725589	0,000478067	0,033209625	Ncan	upregulated
NM_001044276	2,149042053	4,435331856	0,000482164	0,033376253	Rprm	upregulated
NM_001108620	0,444080421	1,360446686	0,000485127	0,033463543	Nup205	upregulated
NM_001047089	0,765139749	1,699534618	0,000492256	0,033698344	Spred1	upregulated
NM_001135798	-0,391975359	-1,312188843	0,000495387	0,033698344	Adck5	downregulated
NM_001134854	0,593159026	1,508546352	0,000507563	0,034387068	Smox	upregulated
NM_017053	0,772949969	1,708760222	0,00051076	0,034387068	Tacr3	upregulated
NM_017049	-0,43708226	-1,353863472	0,000517681	0,034734076	Slc4a3	downregulated
NM_001134545	1,594469558	3,019834651	0,000523818	0,035026313	RGD1565772	upregulated
NM_001004446	-0,521756114	-1,435701786	0,000527711	0,035041653	Smarca2	downregulated
NM_001007612	6,375265425	83,01300273	0,000528575	0,035041653	Ccl7	upregulated
NM_001077640	2,266859541	4,812743518	0,000534742	0,035041653	Gadd45g	upregulated
NM_057134	-0,820950172	-1,766569088	0,00054129	0,035179544	Chrd	downregulated
NM_001013149	-0,470091954	-1,385197754	0,000542015	0,035179544	Mesdc1	downregulated
NM_001191830	-0,873208989	-1,831732698	0,000543519	0,035179544	Dusp23	downregulated
NM_139087	0,99488987	1,992928387	0,000555842	0,035655393	Cgref1	upregulated
NM_001110336	1,281957234	2,431686482	0,000563853	0,035655393	Vegfa	upregulated

NM_001142366	0,50384402	1,417986719	0,000565699	0,035655393	Aqp4	upregulated
NM_001012046	0,906820711	1,874909177	0,000577605	0,036162857	Spry2	upregulated
NM_053522	0,948018781	1,929221483	0,000585235	0,036252502	Rhoq	upregulated
NM_031517	1,107232723	2,154320241	0,000590156	0,036252502	Met	upregulated
NM_001100889	-0,57780622	-1,492577889	0,000591515	0,036252502	Shroom3	downregulated
NM_001106900	-0,595810279	-1,511321169	0,000591945	0,036252502	D3ZUG7_RAT	downregulated
NM_001012121	0,787701193	1,726321529	0,00059756	0,036482709	Prr5	upregulated
NM_001109585	1,843908517	3,589812562	0,000606083	0,036785473	Trim47	upregulated
NM_001109677	-0,8680551	-1,825200684	0,000606261	0,036785473	LOC100125361	downregulated
NM_012870	0,598585897	1,514231617	0,000620093	0,037508961	Tnfrsf11b	upregulated
NM_053802	1,360693841	2,568086578	0,000639611	0,038242741	Tgfbi	upregulated
NM_001109344	1,839771552	3,579533428	0,000651103	0,038787996	RGD1562846	upregulated
NM_001110334	1,292372731	2,449305499	0,000662388	0,039341052	Vegfa	upregulated
NM_012717	0,605543332	1,521551676	0,000665222	0,039390346	Calcrl	upregulated
NM_017275	-0,404374373	-1,323514841	0,000668019	0,039437217	Pnck	downregulated
NM_001014071	0,969954249	1,958778477	0,000680221	0,039849658	Errfi1	upregulated
NM_001044245	0,429878594	1,347120209	0,000687746	0,040049091	Asap1	upregulated
NM_031756	0,414580282	1,332910841	0,00068857	0,040049091	Ggcx	upregulated
NM_001012089	2,700230768	6,499058652	0,000693351	0,04004952	Dusp2	upregulated
NM_022251	1,111617584	2,160877943	0,000693917	0,04004952	Enpep	upregulated
NM_144750	3,001097609	8,006088754	0,000703176	0,040096473	Aspg	upregulated
NM_017061	2,958914691	7,775388119	0,0007033	0,040096473	Lox	upregulated
NM_001013179	-0,644790463	-1,563512185	0,000705702	0,040096473	Hes6	downregulated
NM_198752	-0,32612126	-1,253638385	0,000708317	0,040096473	KIFC2	downregulated
NM_001110099	1,327821008	2,510232525	0,000709359	0,040096473	F1MAG5_RAT	upregulated
NM_134408	0,941300865	1,920258938	0,00070978	0,040096473	Lphn2	upregulated
NM_001030025	1,464879162	2,760403489	0,000727119	0,040934617	Upp1	upregulated
NM_001168630	0,55554126	1,469719929	0,000735335	0,041068065	Cdh9	upregulated
NM_001017479	1,602741309	3,037198727	0,000740467	0,041121024	Tmem100	upregulated
NM_001047094	1,130181401	2,188862608	0,000744749	0,041242307	Spred2	upregulated
NM_053894	-0,665481584	-1,586097638	0,000753799	0,041626199	Jdp2	downregulated
NM_013179	1,103641674	2,148964536	0,00075921	0,041807585	Hcrt	upregulated
NM_001011901	0,390350964	1,310712222	0,00077358	0,042479909	Hsph1	upregulated
NM_001108885	-1,565879284	-2,960578861	0,000776186	0,042504298	D3ZYQ7_RAT	downregulated
NM_133572	-0,575930018	-1,490638078	0,000785487	0,042775335	Cdc25b	downregulated
NM_001108056	0,331895959	1,2586664	0,000793706	0,042984732	Papola	upregulated
NM_001108073	-0,642299084	-1,560814498	0,000800263	0,043220808	Tjp3	downregulated
NM_021838	0,813125657	1,757013963	0,000802536	0,043224816	Nos3	upregulated
NM_133296	1,163181158	2,239506973	0,000805535	0,043267768	Slc6a20	upregulated
NM_001106262	-0,642036013	-1,560529914	0,000809867	0,043365009	Otog	downregulated
NM_057135	1,030882637	2,043273939	0,000816771	0,043514619	Pfkfb3	upregulated
NM_001103354	0,861670561	1,817141242	0,000841537	0,044592374	LOC100125362	upregulated
NM_053903	1,949417159	3,862184698	0,000849364	0,044886141	Efna5	upregulated
NM_031148	0,917878453	1,889334899	0,000871114	0,045405046	Slc20a1	upregulated
NM_030847	1,724720493	3,305160871	0,000873041	0,045405046	Emp3	upregulated
NM_053485	1,56608851	2,96100825	0,000882373	0,0457693	S100a6	upregulated

NM_024483	-0,446491567	-1,362722273	0,000905544	0,046298413	Adra1d	downregulated
NM_001108022	0,755987707	1,688787385	0,000905691	0,046298413	RGD1304624	upregulated
NM_001012743	-0,6565181	-1,576273742	0,000906704	0,046298413	Pip5k1b	downregulated
NM_001106399	-0,825424155	-1,772055949	0,000914821	0,046591888	Spata9	downregulated
NM_001106569	0,791455537	1,730819808	0,00092915	0,04698645	Psmd5	upregulated
NM_019239	-0,312053757	-1,241473749	0,000929698	0,04698645	Mgat3	downregulated
NM_024388	1,122910628	2,177859113	0,00094092	0,047308281	Nr4a1	upregulated
NM_001024290	0,482290452	1,396959748	0,000944744	0,047379369	Gal3st3	upregulated
NM_001107437	-0,371118059	-1,293354766	0,000952532	0,047485759	Jph3	downregulated
NM_001134463	-0,464114754	-1,379470642	0,000954558	0,047485759	RGD1310453	downregulated
NM_001025022	0,781583002	1,719016034	0,00095736	0,047485759	RGD1308059	upregulated
NM_001108743	0,954878926	1,938416952	0,000959117	0,047485759	Elk3	upregulated
NM_001106275	0,687130214	1,61007759	0,000963328	0,047485759	Abhd2	upregulated
NM_001004245	0,860347955	1,815476122	0,000963773	0,047485759	Esam	upregulated
NM_030833	0,783518605	1,721323914	0,000971807	0,047640068	Ifitm2	upregulated
NM_001112742	0,458832133	1,374428762	0,000974175	0,047640068	Gria3	upregulated
NM_001134963	-1,175364907	-2,258499989	0,000988459	0,048151101	Crispld1	downregulated
NM_001008776	1,842819575	3,587104004	0,000989524	0,048151101	Serpina11	upregulated

Supplementary Table 2: List of genes with altered expression levels in the TBI model of epilepsy

Name	logFC	directedFC	PValue	adj.p.value	Gene_id	Direction
NM_053329	-1,821686121	-3,534940962	8,58989E-14	1,52239E-09	Igfals	downregulated
NM_001127635	-2,63863347	-6,227415196	5,22527E-13	4,63038E-09	Zfp9	downregulated
NM_031832	1,906780313	3,749713353	1,06437E-10	3,77275E-07	Lgals3	upregulated
NM_133298	1,205121667	2,305567121	2,36999E-10	0,000000659	Gpnmb	upregulated
NM_001108629	-2,761326217	-6,780192417	2,60279E-10	0,000000659	Ggct	downregulated
NM_001108916	-3,801119765	-13,93962422	1,12393E-09	0,000001245	Bnc1	downregulated
NM_031140	1,178294784	2,263091293	1,25135E-09	1,30457E-06	Vim	upregulated
NM_199093	1,491757363	2,812313377	2,05235E-09	2,02077E-06	Serping1	upregulated
NM_022931	-0,88298823	-1,844191191	4,33701E-09	4,04552E-06	Rims3	downregulated
NM_001007149	-0,78898214	-1,727854984	0,00000001	8,44718E-06	Stau2	downregulated
NM_001107702	1,542316401	2,912617802	0,000000012	9,6805E-06	Fcrl2	upregulated
NM_033237	1,547873322	2,923858151	1,41229E-08	1,04292E-05	Gal	upregulated
NM_001100970	0,934946457	1,91181968	1,94447E-08	1,37847E-05	Aebp1	upregulated
NM_013069	0,814567731	1,758771099	2,25341E-08	1,53605E-05	Cd74	upregulated
NM_017024	0,932275915	1,908284025	4,7875E-08	3,14255E-05	Lcat	upregulated
NM_133624	1,239533604	2,36122186	8,42996E-08	5,33586E-05	Gbp2	upregulated
NM_012618	1,15806293	2,231575984	9,51216E-08	5,81324E-05	S100a4	upregulated
NM_031823	1,586251898	3,002682426	1,1347E-07	6,70342E-05	Wfs1	upregulated
NM_001008884	0,977293471	1,968768488	1,20571E-07	6,89316E-05	RT1-Db1	upregulated
NM_017347	-1,172720159	-2,254363501	3,74602E-07	0,000207471	Mapk3	downregulated

NM_017062	0,685808276	1,608602955	4,34936E-07	0,000233587	Pdlim4	upregulated
NM_134378	1,150771699	2,220326281	5,27711E-07	0,000275077	Sulf1	upregulated
NM_017009	0,789591491	1,728584933	6,34787E-07	0,000321438	Gfap	upregulated
NM_001079942	0,842867316	1,793611354	6,75497E-07	0,000332551	Sema3b	upregulated
NM_031648	0,59701181	1,51258038	8,0821E-07	0,000383319	Fxyd1	upregulated
NM_001101017	1,465422234	2,761442781	9,24307E-07	0,000420039	RT1-Bb	upregulated
NM_001024687	0,70577584	1,631021546	1,14409E-06	0,000506918	Mrc2	upregulated
NM_013179	1,028834607	2,040375393	1,24827E-06	0,000539588	Hcrt	upregulated
NM_080394	-0,56248843	-1,476814302	1,97313E-06	0,000832615	Reln	downregulated
NM_001271051	1,274414591	2,419006408	2,79008E-06	0,001149969	RGD1305645	upregulated
NM_013058	0,669175594	1,590164033	3,45185E-06	0,001364817	Id3	upregulated
NM_012705	0,667435429	1,588247149	3,46537E-06	0,001364817	Cd4	upregulated
NM_012881	1,429307782	2,693174633	4,32592E-06	0,001639027	Spp1	upregulated
NM_001106019	-0,655706061	-1,575386766	4,34657E-06	0,001639027	RGD1560813	downregulated
NM_001136124	1,001346636	2,001867705	4,60571E-06	0,001700564	Ifitm3	upregulated
NM_001004095	1,143098062	2,208547807	6,24308E-06	0,002145193	S100a11	upregulated
NM_144750	1,052860409	2,074639127	6,34709E-06	0,002145193	Aspg	upregulated
NM_012488	0,757005889	1,689979667	6,41512E-06	0,002145193	A2m	upregulated
NM_001102381	2,350448428	5,099827425	7,65562E-06	0,002512602	Nts	upregulated
NM_030868	0,909758855	1,878731444	0,000012865	0,004145573	Nov	upregulated
NM_199101	1,010614038	2,014768441	1,37777E-05	0,00436039	Plekha4	upregulated
NM_012532	1,137433524	2,199893259	1,82254E-05	0,005666823	Cp	upregulated
NM_001009353	0,565478574	1,479878336	1,97525E-05	0,005890488	Pla2g7	upregulated
NM_001011889	0,619307414	1,53613756	2,01001E-05	0,005890488	Cldn9	upregulated
NM_001013086	0,824325858	1,77070743	2,08807E-05	0,005890488	Capg	upregulated
NM_001108696	0,653116663	1,572561744	2,12713E-05	0,005890488	Tp73	upregulated
NM_203410	0,464038475	1,379397708	2,21171E-05	0,006030473	Ifi27	upregulated
NM_022270	0,794281246	1,73421317	2,29377E-05	0,006159478	Slc22a4	upregulated
NM_001025767	0,576702271	1,491436207	2,54463E-05	0,006666231	Blnk	upregulated
NM_019363	0,668032588	1,58890469	2,57667E-05	0,006666231	Aox1	upregulated
NM_012515	0,669803984	1,590856806	2,59533E-05	0,006666231	Tspo	upregulated
NM_001134971	0,434957022	1,35187056	2,70037E-05	0,006836947	Man2b2	upregulated
NM_139193	0,99672608	1,995466529	2,81889E-05	0,00703651	Prlhr	upregulated
NM_212525	0,551512358	1,465621282	2,93842E-05	0,007233005	Tyrobp	upregulated
NM_130427	0,794760067	1,73478884	2,99202E-05	0,007264063	Eya2	upregulated
NM_001013433	0,819627201	1,764949862	3,66853E-05	0,00866898	Arl11	upregulated
NM_017007	-0,47238075	-1,387397077	3,86291E-05	0,009008195	Gad1	downregulated
NM_031970	0,646103232	1,564935538	3,93525E-05	0,009057724	Hspb1	upregulated
NM_053485	1,132125944	2,191814865	4,29755E-05	0,0097648	S100a6	upregulated
NM_030863	0,569430399	1,48393757	4,55562E-05	0,010220156	Msn	upregulated
NM_012563	-0,466077103	-1,38134827	4,75884E-05	0,010542607	Gad2	downregulated
NM_138900	0,777590297	1,714265179	4,83964E-05	0,010589248	C1s	upregulated
NM_031504	0,727112582	1,655322802	5,04823E-05	0,010910941	C4b	upregulated
NM_001108603	-0,639448257	-1,557733307	5,15173E-05	0,010942154	Ptprt	downregulated
NM_001109150	0,474529631	1,389465132	5,71947E-05	0,011925429	Fam181b	upregulated
NM_001191862	0,621461151	1,538432506	5,98102E-05	0,012325767	Flnc	upregulated

NM_139103	0,521529716	1,435476503	6,35605E-05	0,012710322	Cd48	upregulated
NM_001008847	0,615788428	1,532395218	6,84058E-05	0,01332259	RT1-Da	upregulated
NM_017356	-0,533488766	-1,447425171	7,19992E-05	0,013819627	Hpcal1	downregulated
NM_001270594	0,553041206	1,467175249	7,65904E-05	0,014440555	Tmem176b	upregulated
NM_173118	0,493281773	1,407643275	0,000085294	0,015601439	Npc2	upregulated
NM_134350	0,747471766	1,678848173	8,53885E-05	0,015601439	Mx2	upregulated
NM_001106033	0,457603411	1,37325868	8,71982E-05	0,015695638	Efs	upregulated
NM_199499	0,440125272	1,356722129	8,98451E-05	0,015923252	Lgi4	upregulated
NM_172222	0,83468398	1,78346633	0,00009627	0,016892995	C2	upregulated
NM_001271143	0,578244502	1,493031395	9,78781E-05	0,017006799	S1pr3	upregulated
NM_012974	0,436542848	1,353357367	0,00010612	0,01810724	Lamb2	upregulated
NM_138826	0,675921535	1,597616939	0,000106255	0,01810724	Mt1a	upregulated
NM_001246183	1,232319352	2,349443953	0,000114385	0,019307098	Lyzl4	upregulated
NM_030847	0,872626543	1,83099334	0,000116684	0,019509266	Emp3	upregulated
NM_001270558	0,533082566	1,447017697	0,000120481	0,019771094	Aqp4	upregulated
NM_134390	0,518767657	1,432730893	0,000130224	0,020981501	Tmem176b	upregulated
NM_134449	0,558187233	1,47241794	0,000133116	0,021104833	Prkcdbp	upregulated
NM_001135761	0,651070006	1,570332435	0,000133371	0,021104833	Crb2	upregulated
NM_022712	-0,463809644	-1,379178935	0,000161465	0,024662485	Tfrc	downregulated
NM_031059	0,614002295	1,530499205	0,000165546	0,02486415	Msx1	upregulated
NM_001107321	-0,779645066	-1,716708474	0,000168727	0,025128935	Htra4	downregulated
NM_012924	0,747799564	1,679229672	0,000173611	0,025463471	Cd44	upregulated
NM_012760	-1,191690098	-2,284201777	0,000173846	0,025463471	Plagl1	downregulated
NM_001108513	0,537896222	1,451853839	0,000178079	0,025631877	Unc93b1	upregulated
NM_001108227	0,685994876	1,608811028	0,000178535	0,025631877	Wnt10a	upregulated
NM_012665	-0,65815078	-1,5780586	0,000193595	0,027448636	Syt2	downregulated
NM_001134545	0,659952562	1,580030669	0,000211945	0,029346081	Ssc5d	upregulated
NM_001270963	-0,44847155	-1,364593784	0,000213839	0,029378888	Kcnd3	downregulated
NM_030833	0,643641792	1,562267815	0,000216476	0,029512278	Ifitm2	upregulated
NM_001191575	0,733053263	1,662153087	0,000221579	0,029782651	Ptpru	upregulated
NM_001033998	0,654474527	1,574042535	0,000224905	0,029782651	Itgal	upregulated
NM_183330	0,477260878	1,392098097	0,00022506	0,029782651	Ctsz	upregulated
NM_012575	0,444995101	1,361309494	0,000236076	0,03099244	Grin2c	upregulated
NM_001012041	0,604426352	1,520374098	0,000246308	0,031632673	Irf9	upregulated
NM_012580	0,61194716	1,528320544	0,000251857	0,032112684	Hmox1	upregulated
NM_001007002	0,580983932	1,495869098	0,000257027	0,032537787	Mxra8	upregulated
NM_134349	0,791161848	1,730467502	0,000273971	0,034436729	Mgst1	upregulated
NM_001270593	0,50184702	1,416025278	0,000289165	0,036090689	Tmem176b	upregulated
NM_001109410	0,547885033	1,461940943	0,000299201	0,037082134	Trappc6a	upregulated
NM_001106856	0,758151789	1,691322513	0,000304495	0,037393025	Uba7	upregulated
NM_019229	0,382205146	1,303332465	0,00030593	0,037393025	Slc12a4	upregulated
NM_001034011	0,689730079	1,612981709	0,000310926	0,037743397	Sft2d2	upregulated
NM_172030	0,419030722	1,337028967	0,000313558	0,037804019	Entpd2	upregulated
NM_024388	0,509522023	1,423578474	0,000322459	0,038614502	Nr4a1	upregulated
NM_001008830	0,45088526	1,366878737	0,000330546	0,039317174	RT1-A1	upregulated
NM_001107743	0,643838229	1,562480548	0,000358303	0,041456536	Slc43a3	upregulated

NM_013137	0,339879926	1,265651251	0,000363919	0,041456536	Ddr1	upregulated
NM_001142366	0,514831285	1,428827037	0,000364083	0,041456536	Aqp4	upregulated
NM_022667	0,59741873	1,513007071	0,000367501	0,041456536	Slco2a1	upregulated
NM_017125	0,400852464	1,320287816	0,000369223	0,041456536	Cd63	upregulated
NM_001008560	-0,518055388	-1,432023719	0,000370082	0,041456536	Prss35	downregulated
NM_173045	0,593466153	1,508867532	0,000372487	0,041456536	Zc3hav1	upregulated
NM_031776	-0,442659656	-1,359107579	0,000380542	0,041622482	Gda	downregulated
NM_001012357	-0,947363037	-1,928344799	0,000382227	0,041622482	Ccl9	downregulated
NM_021578	0,493777892	1,408127424	0,000382806	0,041622482	Tgfb1	upregulated
NM_001008315	0,42481566	1,342400966	0,000409785	0,043764045	Ltbr	upregulated
NM_012825	0,500647775	1,414848691	0,000416372	0,043764045	Aqp4	upregulated
NM_053018	0,406751201	1,325697119	0,000416701	0,043764045	Cd9	upregulated
NM_001031638	0,819272399	1,764515861	0,000417034	0,043764045	Cd68	upregulated
NM_001137564	0,526774575	1,440704617	0,000432152	0,044648645	Mt2A	upregulated
NM_001012039	0,559784914	1,474049441	0,000433658	0,044648645	Efemp1	upregulated
NM_138524	0,686833747	1,609746761	0,000449665	0,045801178	A3galt2	upregulated
NM_013107	0,537012776	1,450965057	0,000462616	0,046851137	Bmp6	upregulated
NM_001007144	0,460991547	1,376487537	0,000482808	0,048618237	Plin2	upregulated
NM_001005562	0,464770572	1,380097863	0,00050441	0,050222772	Creb3l1	upregulated
NM_001107276	-0,674793784	-1,596368574	0,000509851	0,050480956	Piwil2	downregulated
NM_001106210	0,399223397	1,31879781	0,000530424	0,05222616	Rnaset2	upregulated
NM_022617	0,436620394	1,353430114	0,00053985	0,052799617	Mpeg1	upregulated
NM_001107432	-0,534569491	-1,448509846	0,000555255	0,053073075	Cntnap4	downregulated
NM_001008514	0,842211435	1,792796124	0,000557971	0,053073075	Cldn19	upregulated
NM_019904	0,481544896	1,396238014	0,000558603	0,053073075	Lgals1	upregulated
NM_013154	0,57675467	1,491490377	0,000567306	0,053468842	Cebpd	upregulated
NM_001002807	0,425367902	1,342914916	0,000571788	0,053468842	Clic1	upregulated
NM_012855	0,44283061	1,359268638	0,000595864	0,055290541	Jak3	upregulated
NM_001004261	0,47637561	1,391244139	0,000607785	0,056102976	Pyroxd2	upregulated
NM_175763	0,448674459	1,364785722	0,000663277	0,06032102	Igsf1	upregulated
NM_001108991	0,444944933	1,361262156	0,000663691	0,06032102	Trhde	upregulated
NM_001024275	0,429294271	1,346574706	0,000684652	0,061286334	Rassf4	upregulated
NM_001107503	0,994190294	1,991962232	0,000692109	0,061639392	Cd22	upregulated
NM_001008827	0,405081286	1,324163516	0,00069593	0,061669873	RT1-A1	upregulated
NM_019289	0,41581572	1,334052756	0,000703586	0,06203809	Arpc1b	upregulated
NM_080411	-0,457908619	-1,373549229	0,000709674	0,062265136	Gpr83	downregulated
NM_001107319	-0,421425494	-1,339250184	0,000740797	0,064665429	Unc5d	downregulated
NM_001191970	-0,425246687	-1,342802089	0,000751641	0,064665429	Thsd7a	downregulated
NM_001012205	-0,617105324	-1,53379463	0,000755306	0,064665429	Dpp10	downregulated
NM_001106622	0,502471481	1,416638328	0,000755537	0,064665429	Tapbpl	upregulated
NM_001130569	0,94284664	1,922317502	0,000764027	0,064665429	Mpped1	upregulated
NM_001270559	0,485245875	1,399824414	0,000771519	0,064803972	Aqp4	upregulated
NM_053612	0,429838193	1,347082484	0,000789367	0,065680513	Hspb8	upregulated
NM_133527	0,812205335	1,755893489	0,000802437	0,066456005	Folr1	upregulated
NM_198134	0,594894033	1,510361644	0,000809669	0,06673953	Bst2	upregulated
NM_001100634	-0,869478832	-1,827002784	0,000826859	0,0672221	Tph1	downregulated

NM_001127547	0,385194638	1,306035976	0,00083663	0,067705887	Fbln1	upregulated
NM_053021	0,371281695	1,293501471	0,000864157	0,069615676	Clu	upregulated
NM_001009541	0,513182159	1,427194696	0,000908269	0,072510172	Ier2	upregulated
NM_138521	0,396987472	1,316755488	0,000927359	0,073533577	Ppp1r1b	upregulated
NM_001007691	0,47802385	1,392834506	0,000932406	0,073533577	Prss23	upregulated
NM_001271185	0,73702391	1,666734037	0,000933536	0,073533577	Fzd7	upregulated
NM_001039008	0,3669374	1,289612292	0,000943137	0,073835006	Tmem176a	upregulated
NM_001136229	0,526723089	1,440653203	0,000945695	0,073835006	Trpm4	upregulated
NM_001114405	0,341671641	1,267224068	0,000960547	0,07466567	Ptp4a3	upregulated
NM_173331	0,772340613	1,708038641	0,000979736	0,075824699	Mapk15	upregulated

Supplementary Table 3: Genes with changes in methylation status in introns common to at least two models of epilepsy

Model_1	Model_2	Chromosome	Start	End	Change of methylat	Feature	Gene ID
amygdala stimulation	TBI	chr4	128910356	128911258	increased	introns	Slc25a26
amygdala stimulation	TBI	chr5	161564360	161564871	increased	introns	D4ADI0_RAT
amygdala stimulation	TBI	chr7	21481207	21481543	increased	introns	Nuak1
amygdala stimulation	TBI	chr8	48758435	48758606	increased	introns	Bace1;Dscaml1;D3ZL75_RAT
amygdala stimulation	TBI	chr10	55230217	55230771	increased	introns	Pik3r5
amygdala stimulation	TBI	chr10	103021764	103022737	increased	introns	Slc39a11
amygdala stimulation	TBI	chr11	84850830	84851551	increased	introns	Rtn4r
amygdala stimulation	TBI	chr12	21647993	21648906	increased	introns	F1MAE0_RAT
amygdala stimulation	TBI	chr16	81113554	81114091	increased	introns	Dcun1d2
amygdala stimulation	TBI	chr19	53523019	53523987	increased	introns	Chmp1a
amygdala stimulation	TBI	chr20	13538035	13539191	increased	introns	Cabin1
amygdala stimulation	TBI	chr1	234565077	234565786	decreased	introns	D3ZPY1_RAT
amygdala stimulation	TBI	chr5	34475803	34476750	decreased	introns	Atp6v0d2
amygdala stimulation	TBI	chr13	57449201	57449643	decreased	introns	Cdc73
amygdala stimulation	TBI	chr17	32335793	32336484	decreased	introns	F1M9Z2_RAT
amygdala stimulation	pilocarpine	chr1	206504641	206504882	increased	introns	LOC100365083
amygdala stimulation	pilocarpine	chr2	217740941	217741455	increased	introns	Rwdd3
amygdala stimulation	pilocarpine	chr3	4653515	4654341	increased	introns	Notch1
amygdala stimulation	pilocarpine	chr5	79630442	79631528	increased	introns	Rgs3
amygdala stimulation	pilocarpine	chr5	169508927	169509653	increased	introns	D4ABT6_RAT
amygdala stimulation	pilocarpine	chr11	84366659	84367506	increased	introns	Sept5
amygdala stimulation	pilocarpine	chr3	15197482	15198469	decreased	introns	Lhx6
amygdala stimulation	pilocarpine	chr7	130149173	130149656	decreased	introns	F1LNJ1_RAT

amygdala stimulation	pilocarpine	chr9	101471518	101472051	decreased	introns	Efna5
TBI	pilocarpine	chr5	146252645	146253331	increased	introns	LOC100294508
TBI	pilocarpine	chr6	137928118	137929266	increased	introns	Jag2
TBI	pilocarpine	chr7	117943691	117944463	increased	introns	Gtpbp1
TBI	pilocarpine	chr12	1587524	1589206	increased	introns	D3ZXM4_RAT
TBI	pilocarpine	chr15	24338074	24338910	increased	introns	Peli2
TBI	pilocarpine	chr17	18184095	18185337	increased	introns	Auh
TBI	pilocarpine	chr20	4226927	4227704	increased	introns	F1LWE0_RAT; Stk19; F1LY00_RAT; Agpat1; Rdbp; Ppt2; Prrt1; F1LWU4_RAT; F1LTB0_RAT; Fkbpl; DOM3Z_RAT; Skiv2l; C4b; Cyp21a1; E9PU73_RAT; Rnf5; Atf6b; D3ZWW7_RAT; Egfl8
TBI	pilocarpine	chr1	160172173	160173405	decreased	introns	ENSRNOT00000024998
TBI	pilocarpine	chr3	59406827	59407165	decreased	introns	F1M692_RAT; D4A4B0_RAT; F1M3Y7_RAT; F1M4H3_RAT; Fkbp7; F1LY53_RAT; Plekha3; Prkra; D3ZSE6_RAT
TBI	pilocarpine	chr4	156521363	156522041	decreased	introns	Ninj2
TBI	pilocarpine	chr7	96447841	96449042	decreased	introns	Trib1
TBI	pilocarpine	chr8	114810658	114811627	decreased	introns	Klhl18
TBI	pilocarpine	chr9	101417778	101419340	decreased	introns	Efna5
TBI	pilocarpine	chr12	29955458	29956537	decreased	introns	Tmem132d
TBI	pilocarpine	chr18	27179946	27180423	decreased	introns	Gfra3
TBI	pilocarpine	chr20	4574448	4575354	decreased	introns	Tesb
